# Supplementary material for: Influenza A viral burst size from thousands of infected single cells using droplet quantitative PCR (dqPCR)
Source: PLoS Pathog. 2024 Jul 1;20(7):e1012257. doi: 10.1371/journal.ppat.1012257 (PMC11244780; doi:10.1371/journal.ppat.1012257)
Supplement: S1 Materials and Methods — (PDF) [file ppat.1012257.s001.pdf]

(S1 Materials and Methods) RT-qPCR Template and Primer Sequences. Detection of IAV and host cells was facilitated by reverse transcription quantitative polymerase chain reaction (RT-qPCR) of IAV matrix protein genomic RNA (M gene) and cellular β-actin mRNA. M gene RNA was amplified with forward/reverse primers and a Taqman probe (FAM/BHQ1) that target a highly conserved region shared across IAV strains [1] (S1 Table). β-actin mRNA was amplified with forward/reverse primers and a Taqman probe (Cy5/BHQ2) shown to be robust against multiple eukaryotic cell lines [2] (S1 Table). All primers and probes were purchased from Eurofins Operon as 100 μM stocks. Known concentrations of the template sequences were used as positive controls for RT-qPCR validation throughout this work. The M gene control was first constructed as linear dsDNA (IDT) containing a T7 promoter (underlined), forward and reverse primer binding sites (italicized), and a probe binding site (bold) (S1 Table). M gene RNA was *in vitro* transcribed (IVT) from the ssDNA gblock using the MEGAscript T7 RNA Synthesis Kit (Ambion, #AM1333), and purified over a GE Illustra Sephadex G-50 NICK column. The β-actin control (S1 Table) was constructed as a dsDNA plasmid (pCAG-mGFP-Actin, Addgene #21948), also containing forward and reverse primer binding sites (italicized) and a probe binding site (bold), which was directly amplified by PCR. The concentrations (copies/μL) of template sequence controls in working stocks were quantified with a NanoDrop spectrophotometer. For droplet experiments, template sequence concentrations in copies per drop (cpd) were calculated by multiplying copies/μL by the volume (μL) of a 50 μm (6.54×10^{-5} μL) or 100 μm (or 5.24×10^{-4} μL) diameter drop.

References

1. Shu B, Wu KH, Emery S, Villanueva J, Johnson R, Guthrie E, et al. Design and performance of the CDC real-time reverse transcriptase PCR swine flu panel for detection of 2009 A (H1N1) pandemic influenza virus. J Clin Microbiol. 2011 Jul;49(7):2614–9.

2. Piorkowski G, Baronti C, de Lamballerie X, de Fabritus L, Bichaud L, Pastorino BA, et al. Development of generic Taqman PCR and RT-PCR assays for the detection of DNA and mRNA of β-actin-encoding sequences in a wide range of animal species. J Virol Methods. 2014 Jun;202:101–5.
