# Supplementary material for: Influenza A viral burst size from thousands of infected single cells using droplet quantitative PCR (dqPCR)
Source: PLoS Pathog. 2024 Jul 1;20(7):e1012257. doi: 10.1371/journal.ppat.1012257 (PMC11244780; doi:10.1371/journal.ppat.1012257)
Supplement: S2 Materials and Methods — (PDF) [file ppat.1012257.s002.pdf]

(S2 Materials and Methods) Microfluidic Device Fabrication. Microfluidic devices were fabricated in polydimethylsiloxane (PDMS) (Sylgard 184) using soft lithography. Three device geometries were designed using AutoCAD (Autodesk): a flow-focusing device for encapsulation of infected cells in 100 μm drops, a split-and-merge device used to isolate replicated virus from the host cell and merge with RT-qPCR mix [1], and a flow-based imaging device [2] for drop fluorescence detection. Device master molds were fabricated by patterning SU-8 photoresist (Microchem, SU-8 3050) on silicon wafers (University Wafer, #447) with photolithography. PDMS was prepared at a 10:1 mass ratio of polymer to cross-linking agent and poured onto the device master molds. Air was purged from the uncured PDMS by placing the filled mold in a vacuum chamber for at least 1 hr. The PDMS was cured in an oven at 55 °C for 24 hrs and then removed from the mold with a scalpel. Inlet and outlet ports were punched into PDMS slabs with a 0.75 mm diameter biopsy punch (EMS Rapid-Core, Electron Microscopy Sciences). The split-and-merge device was comprised of two layers bonded together after plasma treatment (Harrick Plasma, PDC-001) for 30 s at medium power and 700 mTorr oxygen pressure. The double-layer PDMS split-and-merge device and single-layer PDMS flow-focusing device were each bonded to 2 × 3 in glass slides (VWR micro slides, #48382-179) after plasma treatment for 60 s at high power and 400 mTorr oxygen pressure. Microelectrodes were embedded in the split-and-merge device by injecting molten solder (Indium Corporation of America, #53307) into the electrode channels, and terminated with a pin terminal (Phoenix Contact, #1945151). Both devices were made hydrophobic by flowing Aquapel (Pittsburgh Glass Works) through the channels, followed by blowing the channels with air passed through a 0.2-μm filter (GVS ABLUO 25 mm, Fisher Scientific) before baking the devices in an oven at 55 °C for 1 hr for further drying.

References

1. Tao Y, Rotem A, Zhang H, Chang CB, Basu A, Kolawole AO, et al. Rapid, targeted and culture-free viral infectivity assay in drop-based microfluidics. Lab Chip. 2015 Sep;15(19):3934–40.

2. Mazutis L, Gilbert J, Ung WL, Weitz DA, Griffiths AD, Heyman JA. Single-cell analysis and sorting using droplet-based microfluidics. Nat Protoc. 2013 May;8(5):870–91.
