# Supplementary material for: Influenza A viral burst size from thousands of infected single cells using droplet quantitative PCR (dqPCR)
Source: PLoS Pathog. 2024 Jul 1;20(7):e1012257. doi: 10.1371/journal.ppat.1012257 (PMC11244780; doi:10.1371/journal.ppat.1012257)
Supplement: S3 Materials and Methods — (PDF) [file ppat.1012257.s003.pdf]

(S3 Materials and Methods) Bulk RT-qPCR. Bulk RT-qPCR reactions were completed using the SuperScript III Platinum One-Step RT-qPCR kit (Invitrogen 11732020) with a final reaction volume of 25 µL. Working stocks of M gene primers and FAM TaqMan probe (S1 Table) were prepared at 25 µM and 10 µM, respectively. The bulk RT-qPCR master mix contained final concentrations of 400 nM primers, 200 nM probe, 0.05 µM ROX reference dye, 2.0 mM $MgSO_4$, 1.0% w/v Tween-20 (Calbiochem 655204-100mL), 0.8 µg/µL BSA (Fisher BP675-1), 1.0 M betaine (Sigma B0300-1VL), 0.32 U/µL SUPERase RNase Inhibitor (Invitrogen AM2694), and 1 µL of SuperScript III RT/Platinum Taq mix (Invitrogen 11732020). The additives (Tween-20, BSA, betaine) increase stability of dqPCR reactions, as determined by our previously published protocol [1]. Thermocycling was performed using a standard qPCR machine (QuantStudio 7, Applied Biosystems) with the following conditions: 1 cycle for 30 mins at 60 °C, 1 cycle for 2 mins at 95 °C, and 40 cycles between 15 s at 95 °C and 1 min at 60 °C. Reference amplification curves for the bulk RT-qPCR assay were constructed from a dilution series of M gene IVT RNA (S2 Table).

References

1. Loveday EK, Zath GK, Bikos DA, Jay ZJ, Chang CB. Screening of Additive Formulations Enables Off-Chip Drop Reverse Transcription Quantitative Polymerase Chain Reaction of Single Influenza A Virus Genomes. Anal Chem. 2021 Mar;93(10):4365–73.
