# Supplementary material for: Influenza A viral burst size from thousands of infected single cells using droplet quantitative PCR (dqPCR)
Source: PLoS Pathog. 2024 Jul 1;20(7):e1012257. doi: 10.1371/journal.ppat.1012257 (PMC11244780; doi:10.1371/journal.ppat.1012257)
Supplement: S4 Materials and Methods — (PDF) [file ppat.1012257.s004.pdf]

(S4 Materials and Methods) Droplet Quantitative PCR (dqPCR) of Isolated Progeny Viruses. A multiplexed dqPCR assay was used to amplify both IAV M gene RNA and cellular β-actin mRNA in drops containing progeny virus. The dqPCR master mix contained the same reagent concentrations as the bulk RT-qPCR, except for an increased concentration of ROX reference dye at 0.15 µM. Working stocks of the M gene primers and FAM TaqMan probe, as well as the β-actin primers and Cy5 TaqMan probe (S1 Table), were prepared at concentrations of 25 µM and 10 µM, respectively. Droplets containing infected cells were re-injected into a split-and-merge device [1] at a flow rate of 500 µL/hr with oil (3.0 wt% RAN surfactant in HFE 7500) flowing at 2000 µL/hr to space them apart. In the device, a split junction was used to isolate progeny viruses from the host cell. Approximately 1/8th volume of the drop containing progeny virus was split and sent to the merge junction. To separate split drops before the merge junction, a second spacer oil (3.0 wt% RAN surfactant in HFE 7500) was injected into the device at a flow rate of 500 µL/hr. At the merge junction, the RT-qPCR master mix was injected into the split drop at a flow rate of 438 µL/hr. Merging was facilitated by an embedded microelectrode that provided a constant 25 kHz, 200 V square wave signal to destabilize the drop interface and allow merging with the RT-qPCR mix. The microelectrode was connected to a high voltage amplifier (Trek Model, #2220-CE) controlled by a custom LabVIEW program. Merged drops were collected in PCR tubes for 2.5 min each to sample approximately 20 µL of drops. PCR tubes were stored on a cold block until ready for thermocycling. Drops were thermocycled in a standard qPCR machine (QuantStudio 7, Applied Biosystems) with the following conditions: 1 cycle for 30 min at 60 °C, 1 cycle for 2 min at 95 °C, and 40 cycles between 15 sec at 95 °C and 1 min at 60 °C. Drops were removed from the thermocycler at cycle numbers N = 1 and N = 40, to measure baseline and terminal fluorescence, as well as multiple intermediate cycle numbers falling within the exponential to linear region of PCR amplification curves. Reference amplification curves for the dqPCR assay were constructed by encapsulating 10^6 copies/µL of M gene IVT RNA and β-actin plasmid (S1 Table) into 50 µm drops using a flow-focusing device, which corresponds to 10^2 cpd of M gene and β-actin. Drops containing M gene or β-actin template sequence controls were collected in 3 mL syringes for 36 min to generate 600 µL of drops and kept on ice until thermocycling with the above conditions. The method for constructing reference amplification curves from dqPCR of template sequence controls is further described in **S8 Materials and Methods.**

References

1. Tao Y, Rotem A, Zhang H, Chang CB, Basu A, Kolawole AO, et al. Rapid, targeted and culture-free viral infectivity assay in drop-based microfluidics. Lab Chip. 2015 Sep;15(19):3934–40.
