# Supplementary material for: Influenza A viral burst size from thousands of infected single cells using droplet quantitative PCR (dqPCR)
Source: PLoS Pathog. 2024 Jul 1;20(7):e1012257. doi: 10.1371/journal.ppat.1012257 (PMC11244780; doi:10.1371/journal.ppat.1012257)
Supplement: S5 Materials and Methods — (PDF) [file ppat.1012257.s005.pdf]

(S5 Materials and Methods) Drop Fluorescence Imaging. As a positive control, thermocycled drops were imaged to capture five fields of view on an inverted epifluorescence microscope (Nikon Ti2-E) at 10× magnification (NA 0.3). Brightfield and fluorescence images were captured with a sCMOS camera (Hamamatsu, ORCA-Flash 4.0 v3) for each reporter fluorescence, FAM TaqMan for M gene RNA (FITC channel), Cy5 TaqMan probe for β-actin RNA (Cy5 channel), as well as for the ROX reference dye (Texas Red channel). Drop fluorescence intensities are reported as ΔR_N, which is the ratio of the M gene (FAM) or β-actin (Cy5) reporter florescence to the reference dye fluorescence (ROX) at each sampled cycle number (N), normalized to the baseline at $N = 1$. Here, $R_N = (F_{N,\ FAM}\ or\ F_{N,\ Cy5})/F_{N,\ ROX}$ and $\Delta R_N = R_N - R_{N=1}$.
