# Supplementary material for: Influenza A viral burst size from thousands of infected single cells using droplet quantitative PCR (dqPCR)
Source: PLoS Pathog. 2024 Jul 1;20(7):e1012257. doi: 10.1371/journal.ppat.1012257 (PMC11244780; doi:10.1371/journal.ppat.1012257)
Supplement: S6 Materials and Methods — (PDF) [file ppat.1012257.s006.pdf]

(S6 Materials and Methods) Drop Flow-based Fluorescence Detection. Thermocycled drops were injected into a continuous-flow microfluidic detection device [1] at a flowrate of 200 µL/hr, along with fluorinated spacer oil (HFE 7500) injected at a flowrate of 800 µL/hr. Drop fluorescence intensity (ΔR_N) was detected in high-throughput using a Nikon Ti-U inverted microscope custom-modified with three lasers, a set of dichroic mirrors, and two photomultiplier tubes (PMTs, Hamamatsu H10723-20). The beams of the 22 mW 488 nm laser (Thorlabs MCLS1), 25 mW 561 nm laser (Cobolt Jive), and 20 mW 642 nm laser (Thorlabs MCLS1) were aligned and coupled into the backport of the microscope where they were focused to a spot by the 40× objective (NA 0.60). The flowing of drops across the laser spot resulted in fluorescence detection by three PMTs split into three channels using dichroic and bandpass filters, Ch1: 520/40 nm (green), Ch2: 600/40 nm (red), and Ch3: 670/10 nm (far-red). A field programmable gate array (FPGA, National Instruments NI-7852R) was used to control the PMT gains and record fluorescence measurements using LabVIEW 2015. A custom MATLAB (R2020a) script was used to process and analyze the drop fluorescence detection data.

References

1. Mazutis L, Gilbert J, Ung WL, Weitz DA, Griffiths AD, Heyman JA. Single-cell analysis and sorting using droplet-based microfluidics. Nat Protoc. 2013 May;8(5):870–91.
