# Supplementary material for: Influenza A viral burst size from thousands of infected single cells using droplet quantitative PCR (dqPCR)
Source: PLoS Pathog. 2024 Jul 1;20(7):e1012257. doi: 10.1371/journal.ppat.1012257 (PMC11244780; doi:10.1371/journal.ppat.1012257)
Supplement: S7 Materials and Methods — (PDF) [file ppat.1012257.s007.pdf]

(S7 Materials and Methods) Cycle Threshold (*Ct*) Method of Creating Standard Curves for RT-qPCR. In standard bulk RT-qPCR, the nucleic acid template concentration of an unknown sample is determined by comparing its cycle threshold (*Ct*) value to a standard curve made from a dilution series of known template concentrations. The standard curve follows Eq. S1:

$$\boldsymbol{Ct = m * log_{10}(C_{RNA}) + b}$$

(Eq. S1)

Cycle threshold (*Ct*) is the PCR cycle number at which template amplification fluorescence rises above background fluorescence, C_{RNA} is the known template concentration, *m* is the slope of the *Ct* versus $\log_{10}(C_{RNA})$ line, and *b* is the y-intercept of the line.

This method assumes that PCR efficiency at the *Ct* value (E_{Ct}) is constant, regardless of starting template concentration. An optimized PCR assay should have an E_{Ct} between 90 – 110% for accurate quantification of template concentration. E_{Ct} is calculated using *m* of the *Ct* versus $\log_{10}(C_{RNA})$ line, as shown in Eq. S2:

$$\boldsymbol{E_{Ct} = 10^{\frac{-1}{m}} - 1}$$

(Eq. S2)

For an ideal assay with E_{Ct} = 100%, the Ct values of a 10-fold dilution series are spaced approximately 3.3 cycles apart ($m \cong -3.3$).
