# Supplementary material for: Influenza A viral burst size from thousands of infected single cells using droplet quantitative PCR (dqPCR)
Source: PLoS Pathog. 2024 Jul 1;20(7):e1012257. doi: 10.1371/journal.ppat.1012257 (PMC11244780; doi:10.1371/journal.ppat.1012257)
Supplement: S8 Materials and Methods — (PDF) [file ppat.1012257.s008.pdf]

**(S8 Materials and Methods) Sigmoidal Curve Fitting using PCR Efficiency (SCF-E).** To track RNA amplification during dqPCR, we reconstructed continuous RT-qPCR amplification curves from discontinuous drop fluorescence measurements, as shown in Fig 2A. Amplification curves can be fit to discontinuous drop fluorescence measurements using a sigmoidal curve fitting (SCF) model. Here, we employ SCF that relates reaction efficiency to fluorescence intensity to accurately model the shape of RT-qPCR amplification curves. This method is called "Sigmoidal Curve Fitting Using PCR Efficiency," or SCF-E. We used SCF-E to generate reference amplification curves of a known RNA template concentration during dqPCR.

RT-qPCR amplification curves can have an asymmetric shape due to a decrease in PCR reaction efficiency after each cycle number. At early cycles, PCR efficiency is near 100%, relating to an exact doubling of nucleic acid template sequence at each cycle, and approaches 0% at later cycles as PCR reagents become consumed [1]. This relationship leads to a steeper slope at the start of the curve during exponential amplification and a flatter slope near the final plateau phase [2]. To model asymmetric amplification curves from fluorescence data alone, SCF methods typically set a limit on the number of cycles that are fit [3] or add asymmetric fitting terms [4] [5]. Alternative methods first model PCR efficiency with a sigmoidal [6] or bilinear [2] equation, and use this to construct a fluorescence amplification curve. In our SCF-E model, asymmetric RT-qPCR amplification curves are fit to experimental fluorescence measurements using a four-parametric sigmoid function that includes a PCR efficiency parameter, $E_N$, as shown in Eq. S3:

$$\boldsymbol{E_N = \frac{E_{max}}{1 + e^{\left(\frac{N - N_{0.5}}{k}\right)}} + E_{min}}$$

**(Eq. S3)**

$E_N$ is the PCR efficiency at cycle $N$, $E_{max}$ is the maximum PCR efficiency in the range of 0.9 to 1, $E_{min}$ is the minimum PCR efficiency in the range of 0 to 0.1, and $N_{0.5}$ is the cycle at which $E_N$ equals 0.5. The parameter $k$ determines the shape of the amplification curve, with larger values flattening the curve and smaller values steepening the curve. An example of the PCR efficiency curve is illustrated in S2 Fig (blue dashed curve).

During RT-qPCR, RNA amplification is detected with a complementary fluorescent probe during thermocycling. The rate of fluorescence accumulation during RT-qPCR is determined by the reaction efficiency, as described by Eq. S4:

$$\boldsymbol{F_{N+1} = F_N(1 + E_N)}$$

**(Eq. S4)**

$F_N$ is the fluorescence intensity of the amplification curve at cycle $N$, and $F_{N+1}$ is the fluorescence intensity of the amplification curve at cycle $N+1$. The fluorescence amplification curve is also illustrated in S2 Fig (blue solid curve).

Eqs. S3 and S4 are combined to yield Eq. S5, the **SCF-E model**:

$$\boldsymbol{F_{N+1} = F_N\left(1 + \frac{E_{max}}{1 + e^{\left(\frac{N-N_{0.5}}{k}\right)}} + E_{min}\right)}$$

**(Eq. S5)**

To generate an RT-qPCR amplification curve of $F_N$ at all PCR cycle numbers ($N$ = 1 to 40), we vary the efficiency parameters in Eq. S3 ($E_{max}$, $E_{min}$, $N_{0.5}$, and $k$) to find the best fit to our experimental $F_N$ values, measured at only a few PCR cycle numbers. Thirty values of each of the four efficiency parameters are used to test $8.1 \times 10^5$ ($30^4$) curve fits. The set of parameters that yield the best fit (highest $R^2$ value) to experimental $F_N$ measurements are used to construct the RT-qPCR amplification curve. An initial input for $F_N$ of any non-zero integer is chosen for the fit; here, we used $F_N = 1$. A calibration factor ($CF$), shown in Eq. S6, is used to normalize $F_N$ at $N$ = 40 in the SCF-E model curve ($F_{endpoint,\ model}$) to the experimentally measured $F_N$ at $N$ = 40 ($F_{endpoint,\ measured}$):

$$\boldsymbol{CF = F_{endpoint,measured}/F_{endpoint,model}}$$

**(Eq. S6)**

References

1. Gevertz JL, Dunn SM, Roth CM. Mathematical model of real-time PCR kinetics. Biotechnol Bioeng. 2005 Nov;92(3):346–55.

2. Lievens A, Van Aelst S, Van den Bulcke M, Goetghebeur E. Enhanced analysis of real-time PCR data by using a variable efficiency model: FPK-PCR. Nucleic Acids Res. 2012 Jan;40(2):e10.

3. Rutledge RG. Sigmoidal curve-fitting redefines quantitative real-time PCR with the prospective of developing automated high-throughput applications. Nucleic Acids Res. 2004 Dec;32(22):e178.

4. Tellinghuisen J, Spiess AN. qPCR data analysis: Better results through iconoclasm. Biomol Detect Quantif. 2019 Mar;17:100084.

5. Spiess AN, Feig C, Ritz C. Highly accurate sigmoidal fitting of real-time PCR data by introducing a parameter for asymmetry. BMC Bioinformatics. 2008 Apr;9:221.

6. Alvarez MJ, Vila-Ortiz GJ, Salibe MC, Podhajcer OL, Pitossi FJ. Model based analysis of realtime PCR data from DNA binding dye protocols. BMC Bioinformatics. 2007 Mar;8:85.
