# Supplementary material for: Influenza A viral burst size from thousands of infected single cells using droplet quantitative PCR (dqPCR)
Source: PLoS Pathog. 2024 Jul 1;20(7):e1012257. doi: 10.1371/journal.ppat.1012257 (PMC11244780; doi:10.1371/journal.ppat.1012257)
Supplement: S9 Materials and Methods — (PDF) [file ppat.1012257.s009.pdf]

(S9 Materials and Methods) Amplification Curve Library (ACL) Method of Constructing Standard Curves for RT-qPCR. We generated a high-resolution dilution series of 1000 RNA template concentrations to construct standard curves for conversion of drop fluorescence to RNA concentration. This is performed by first generating a single reference amplification curve for a known IAV M gene RNA concentration (10^2 cpd) (Fig 2A), that is fit using the SCF-E model (Eq. S5). The reference amplification curve is then translated along its *x*-axis by varying the SCF-E $N_{0.5}$ parameter 1000 times, between cycles 1 to 40, to build what we call an "Amplification Curve Library" (ACL) of 1000 virtual curves (Fig 2B, plot with subset of 10 curves). The ACL is then used to construct a virtual standard curve from a single PCR cycle number (Fig 2C). This method for generating standard curves is described in detail below in three sections.

(1) Translating a Single SCF-E Reference Curve into 1000 Virtual Amplification Curves: The ACL is constructed by expanding a single reference amplification curve, fit with Eq. S5, into 1000 virtual curves. This is done by substituting $N_{0.5,\ ref}$, fit from Eq. S5, with $N_{0.5,\ virt}$, to make Eq. S7:

$$\boldsymbol{F_{N+1} = F_N\left(1 + \frac{E_{max}}{1 + e^{\left(\frac{N - N_{0.5,virt}}{k}\right)}} + E_{min}\right)}$$

(Eq. S7)

The translation of the reference curve into virtual curves using Eq. S7, is illustrated in S3 Fig. Here, F_N and E_N are plotted on the left and right *y*-axes, *N* on the *x*-axis (S3 Fig). The solid blue curve is an example of a reference curve fit using the SCF-E model (Eq. S5) and the dashed blue curve is the corresponding PCR efficiency curve, as previously described (S2 Fig). The reaction efficiency parameters in Eq. S7 (E_{max}, E_{min}, *k*) are taken from the reference curve fit (Eq. S5) and are constant across all the virtual curves. In the ACL, each virtual curve is defined by a different cycle number at which PCR reaction efficiency equals 0.5 ($N_{0.5,\ virt}$). We input 1000 evenly spaced $N_{0.5,\ virt}$ values, between cycle numbers $N = 1$ to 40, into Eq. S7 to produce 1000 unique virtual curves. Two virtual curves are displayed in S3 Fig, to illustrate that $N_{0.5,\ virt} < N_{0.5,\ ref}$ (pink curve) is associated with virtual RNA concentrations ($C_{RNA,\ virt}$) greater than the reference ($C_{RNA,\ ref}$). Conversely, if $N_{0.5,\ virt} > N_{0.5,\ ref}$ (yellow curve) then $C_{RNA,\ virt} < C_{RNA,\ ref}$.

(2) Calculating RNA Template Concentration of the Virtual Amplification Curves: $C_{RNA,\ virt}$ is determined by its *x*-axis distance from $C_{RNA,\ ref}$. Since the *x*-axis of the ACL is cycle number, we can relate the virtual and reference curves using the cycle numbers at which PCR efficiency equals 0.5, or $N_{0.5,\ virt}$ and $N_{0.5,\ ref}$. The $N_{0.5,\ virt}$ is a fixed array of 1000 intervals between cycle numbers N = 1 to 40, as previously mentioned (Eq. S7), while $N_{0.5,\ ref}$ is a single cycle number on the reference curve (fit from Eq. S5). The relationship between an amplification curve's $N_{0.5}$ value and its C_{RNA} value yields a line that follows Eq. S8 below, which is analogous to the *Ct* method (Eq. S1), with $N_{0.5}$ used in place of *Ct*.

$$\boldsymbol{N_{0.5} = m * log_{10}(C_{RNA}) + b}$$

(Eq. S8)

We expand this equation for $N_{0.5,\ virt}$ and $N_{0.5,\ ref}$ and take the difference with Eq. S9:

$$\boldsymbol{N_{0.5,virt} - N_{0.5,ref} = \left[m * log_{10}(C_{RNA,virt}) + b\right] - \left[m * log_{10}(C_{RNA,ref}) + b\right]}$$

(Eq. S9)

Eq. S9 is rearranged in Eqs. S10-11 in order to solve for $C_{RNA,\ virt}$ in Eq. 12:

$$\boldsymbol{log_{10}(C_{RNA,virt}) = log_{10}(C_{RNA,ref}) + \frac{N_{0.5,virt} - N_{0.5,ref}}{m}}$$

(Eq. S10)

$$\boldsymbol{f = log_{10}(C_{RNA,ref}) + \frac{N_{0.5,virt} - N_{0.5,ref}}{m}}$$

(Eq. S11)

$$\boldsymbol{C_{RNA,virt} = 10^{f}}$$

(Eq. S12)

Note that the value of $C_{RNA,\ virt}$ varies with *m* from Eq. S8 when used in Eq. S11. To calculate *m*, Eq. S2 is rearranged into Eq. S13:

$$\boldsymbol{m = \frac{1}{log_{10}(E_N + 1)}}$$

(Eq. S13)

m is dependent upon E_N of the reference curve at a single cycle number, calculated with Eq. S3. As E_N increases, *m* decreases, and the range of $C_{RNA,\ virt}$ values in the ACL widens (S4 Fig). To determine the E_N constant used to calculate *m* in Eq. S13, we empirically compared standard curves from libraries built with

different E_N values **(S3 Results)**. Briefly, E_N corresponding to $N = N_{0.5} - 3$ was used to determine virtual RNA concentration for all ACLs used in this work.

(3) Constructing a Standard Curve from the ACL: To determine the RNA concentrations of unknown samples based on their fluorescence intensity, we utilize the ACL (S5A Fig) to construct standard curves that relate C_{RNA} to F_N (S5B Fig). The standard curve is constructed by selecting one PCR cycle number in the ACL (S5A Fig, vertical line) and relating F_N at that cycle number to $C_{RNA,\ virt}$ of curves intersecting the line. The usable region of the standard curve (S5B Fig, grey box) generally corresponds to the exponential to linear region of the virtual amplification curves (S5A Fig, grey box). Outside of these regions, F_N has either not risen above baseline fluorescence at early cycle numbers or has reached saturation at late cycle numbers. Threshold values are set for F_N to define these usable regions, where the upper threshold is set at 1 standard deviation below the F_N at N = 40, and the lower threshold is set as the 99th percentile of the F_N at N = 1, similar to the thresholds generally set by commercial qPCR machines.
