# Supplementary material for: Influenza A viral burst size from thousands of infected single cells using droplet quantitative PCR (dqPCR)
Source: PLoS Pathog. 2024 Jul 1;20(7):e1012257. doi: 10.1371/journal.ppat.1012257 (PMC11244780; doi:10.1371/journal.ppat.1012257)
Supplement: S1 Results — (PDF) [file ppat.1012257.s010.pdf]

(S1 Results) Comparing M Gene Abundance During Bulk and Drop Infections. To demonstrate that encapsulation in drops does not affect IAV infection dynamics [1] during our burst size experiments, we compared the M gene RNA abundance in drop and bulk infections using a bulk RT-qPCR assay (S1 Fig). For bulk infections, we collected both the supernatant and cellular monolayer from each well and prepared them for bulk RT-qPCR. The 0 hpi bulk samples were frozen overnight at -80 °C while the 18 hpi bulk samples were incubated overnight at 37 °C. Preparation of drop infections for bulk RT-qPCR involved freezing a 300 μL sample of drops at -80 °C for 30 mins to break the emulsion. 200 μL of the broken emulsion was collected for further processing. Collected bulk and drop infections were clarified by centrifugation at 500 × *g* for 5 min, and the resulting supernatant was sampled for RT-qPCR targeting the IAV M gene. For both strains, there was greater virus production in drops compared to bulk infection (S1 Fig, 18 hpi), when measured with a two-sample Student's t-test ($p << 0.05$). The relative increase in virus production, between drop and bulk infection, was 6.8× for H3N2 and 2.4× H1N1 infections. On average, H3N2 production in drops was 5.0× greater than H1N1 ($p << 0.05$).

References

1. Loveday EK, Sanchez HS, Thomas MM, Chang CB. Single-Cell Infection of Influenza A Virus Using Drop-Based Microfluidics. Microbiol Spectr. 2022 Sep;e0099322.
