# Supplementary material for: Influenza A viral burst size from thousands of infected single cells using droplet quantitative PCR (dqPCR)
Source: PLoS Pathog. 2024 Jul 1;20(7):e1012257. doi: 10.1371/journal.ppat.1012257 (PMC11244780; doi:10.1371/journal.ppat.1012257)
Supplement: S2 Results — (PDF) [file ppat.1012257.s011.pdf]

(S2 Results) Validating SCF-E with Bulk RT-qPCR of IAV M Gene. Using bulk RT-qPCR amplification of the IAV M gene across five orders of magnitude (S2 Table, 10 fold dilutions of 2.62×10^4 to 2.62×10^9 copies/µL), we validated the use of our SCF-E model (S6A Fig) compared to the standard SCF model (S6B Fig) [1] [2]. SCF-E fit parameters are listed in S3 Table. The SCF-E model provided a better fit across all six RNA concentrations compared to SCF (S3 Table, R^2 of SCF-E vs. SCF). The differences in fit were most pronounced in the exponential and plateau regions of RT-qPCR amplification curves, as demonstrated in S6C Fig for 2.62×10^7 copies/µL (magnified insets).

References

1. Rutledge RG. Sigmoidal curve-fitting redefines quantitative real-time PCR with the prospective of developing automated high-throughput applications. Nucleic Acids Res. 2004 Dec;32(22):e178.

2. Rutledge RG, Stewart D. A kinetic-based sigmoidal model for the polymerase chain reaction and its application to high-capacity absolute quantitative real-time PCR. BMC Biotechnol. 2008 May;8:47.
