# Supplementary material for: Influenza A viral burst size from thousands of infected single cells using droplet quantitative PCR (dqPCR)
Source: PLoS Pathog. 2024 Jul 1;20(7):e1012257. doi: 10.1371/journal.ppat.1012257 (PMC11244780; doi:10.1371/journal.ppat.1012257)
Supplement: S3 Results — (PDF) [file ppat.1012257.s012.pdf]

(S3 Results) Setting a PCR Efficiency Constant (E_N) for the ACL. To calculate $C_{RNA, virt}$ in the ACL, we needed to determine the E_N constant used to calculate m in Eq. S13. E_N is the PCR efficiency at each cycle number (N = 1 to 40) in the SCF-E reference curve (Eq. S5) and m is the slope of the C_{RNA} vs. $N_{0.5}$ line (Eq. S8). To determine which of the 40 possible reference curve E_N values to use in Eq. S13, we constructed ACLs with different E_N values and compared the dynamic range of their standard curves (S4 Table). A schematic of the iterative process for determining E_N at different N values is displayed in S7 Fig.

We first tested E_N at $N = N_{0.5} – 0$, using a SCF-E reference curve of 10^7 copies/μL of IAV M gene RNA amplified by bulk RT-qPCR. This yielded E_N = 0.514 and corresponding m = 5.55 (S4 Table). These values were used to build an ACL, from which we constructed a standard curve at cycle 20. The standard curve was used to convert F_N to C_{RNA} for a known dilution series of IAV M gene RNA (S2 Table, 3 replicates of 21 concentrations between 2.62 × 10^4 to 2.62 × 10^9 copies/μL) amplified by bulk RT-qPCR. To determine the accuracy of the standard curve, we performed a linear regression between measured ($C_{RNA, ACL}$) and expected ($C_{RNA, expected}$) RNA concentrations (S8 Fig, $N = N_{0.5} – 0$, R^2 = 0.886). Samples in the dilution series with a $C_{RNA, ACL}$ value within 2-fold change of $C_{RNA, expected}$ were included in the dynamic range of the standard curve (S4 Table). For E_N at $N = N_{0.5} – 0$, the dynamic range of the cycle 20 standard curve was small, a value of 1.60 $\log_{10}$ RNA copies/μL (S4 Table, dynamic range of 3.28 × 10^6 to 1.31 × 10^8 copies/μL). Thus, to widen the dynamic range, we tested higher E_N values at five cycles preceding $N_{0.5}$ (S4 Table, $N = N_{0.5} – 1$, $N = N_{0.5} – 2$, $N = N_{0.5} – 3$, $N = N_{0.5} – 4$, and $N = N_{0.5} – 5$). The ACL standard curve with the widest dynamic range and highest degree of accuracy (R^2) between $C_{RNA, ACL}$ and $C_{RNA, expected}$ was built with $N = N_{0.5} – 3$, where E_N = 0.745. For this ACL, the dynamic range of the standard curve was 4.70 $\log_{10}$ RNA copies/μL (S4 Table, dynamic range of 2.62 × 10^4 to 1.31 × 10^9 copies/μL) with R^2 = 0.990 between $C_{RNA, ACL}$ and $C_{RNA, expected}$. Therefore, the E_N corresponding to $N = N_{0.5} – 3$ was used to build all amplification curve libraries in this work.
