# Supplementary material for: Influenza A viral burst size from thousands of infected single cells using droplet quantitative PCR (dqPCR)
Source: PLoS Pathog. 2024 Jul 1;20(7):e1012257. doi: 10.1371/journal.ppat.1012257 (PMC11244780; doi:10.1371/journal.ppat.1012257)
Supplement: S4 Results — (PDF) [file ppat.1012257.s013.pdf]

(S4 Results) Validating ACL Standard Curves for Multiple PCR Cycle Numbers. To pool $C_{RNA, ACL}$ values sampled at multiple PCR cycle numbers, we validated that ACL standard curves converted F_N to $C_{RNA, ACL}$ within an acceptable error range of 2-fold change from $C_{RNA, expected}$, at any cycle number. This was done using a known dilution series of IAV M gene RNA amplified by bulk RT-qPCR (S2 Table, 3 replicates of 21 concentrations between 2.62×10^4 to 2.62×10^9 copies/μL). Each ACL was built from a SCF-E reference curve of 2.62×10^7 M gene copies/μL. Standard curves were constructed at ACL N = 17 to 23, and used to converted F_N to $C_{RNA, ACL}$ for the above dilution series. These standard curve cycle numbers were chosen as they typically fall in the linear region of our M gene reference curves. To determine how well $C_{RNA, ACL}$ matched to $C_{RNA, expected}$, we performed a linear regression on these values (S9 Fig). Samples in the dilution series whose $C_{RNA, ACL}$ fell within a 2-fold change of $C_{RNA, expected}$ were included in the dynamic range for each standard curve (S5 Table). We found a wide dynamic range (S5 Table, dynamic ranges from 4.00 to 4.70) and a strong linear relationship between the $C_{RNA, ACL}$ and $C_{RNA, expected}$ (S9 Fig, R^2 ranging from 0.800 to 0.991) for standard curves from all tested cycle numbers.

We validate that the performance of ACL standard curves at different cycle numbers is comparable to that of *Ct* standard curves used in typical bulk RT-qPCR. With a *Ct* standard curve, we convert F_N to $C_{RNA, Ct}$ for the same dilution series of IAV M gene RNA (S2 Table). This conversion resulted in a wide dynamic range of 5 orders of magnitude and a strong linear response between $C_{RNA, Ct}$ and $C_{RNA, expected}$ of R^2 = 0.991 (S9 Fig, under *Ct* plot; S5 Table under *Ct* method).

To further quantify the variability between standard curves from different ACL cycle numbers, we calculated the coefficient of variation (*CV*) of $C_{RNA, ACL}$ replicates (S10A Fig) and percent difference (*%*) between $C_{RNA, ACL}$ and $C_{RNA, expected}$ (S10B Fig). *CV* (%) is the ratio of the standard deviation to the mean $C_{RNA, ACL}$, and is used to describe variability *within* replicates of the same M gene RNA concentration. We found non-linear relationships (S10A Fig, black dotted lines with R^2 values < 0.28) between *CV* (%) and M gene RNA concentration at all sampled cycle numbers. We also calculated the percent difference (*%*) to describe variability *between* $C_{RNA, ACL}$ and $C_{RNA, expected}$ using Eq. S14:

$$\% \, \boldsymbol{difference} = \frac{\boldsymbol{log}\left(\boldsymbol{C_{RNA,ACL}}\right) - \boldsymbol{log}\left(\boldsymbol{C_{RNA,expected}}\right)}{\boldsymbol{log}\left(\boldsymbol{C_{RNA,expected}}\right)} * \mathbf{100}$$

(Eq. S14)

Again, we found a non-linear relationship across RNA concentrations (S10B Fig, black dotted lines with R^2 values < 0.36). We note that variability in conversion with ACL standard curves is comparable to that of the *Ct* method, using *CV* (*%*) (S10A Fig, *Ct*, $R^2 = 0.02$) and percent difference (*%)* (S10B Fig, *Ct*, $R^2 = 0.25$).
