# Supplementary material for: Influenza A viral burst size from thousands of infected single cells using droplet quantitative PCR (dqPCR)
Source: PLoS Pathog. 2024 Jul 1;20(7):e1012257. doi: 10.1371/journal.ppat.1012257 (PMC11244780; doi:10.1371/journal.ppat.1012257)
Supplement: S5 Results — (PDF) [file ppat.1012257.s014.pdf]

(S5 Results) Validating ACL Standard Curves for Multiple Template Sequences. ACL standard curves were also validated for two additional template sequences, the human MYCN gene DNA and soybean Lectin endogene DNA. These were compared to the previously discussed M gene dataset (S11A Fig). The M gene dataset consisted of 21 RNA concentrations (S2 Table, 3.28×10^3 to 2.62×10^9 copies/µL) used in previous positive control and validation experiments. The human MYCN gene and soybean Lectin endogene DNA datasets were taken from previously published studies on the evaluation of qPCR curve analysis methods [1] and enhanced analysis of qPCR data using a variable efficiency model [2]. The MYCN dataset consisted of four DNA concentrations (ranging from 1.88×10^0 to 1.88×10^3 copies/µL) quantified with a SYBR Green qPCR assay. The Lectin dataset consisted of five dilutions (ranging from 6.4×10^0 to 4.0×10^3 copies/µL), also quantified with a SYBR Green qPCR assay. For each of the three template sequences, we constructed an ACL library from reference curves of 10^7 copies/µL for the M gene, 1875 copies/µL for the MYCN gene, and 800 copies/µL for the Lectin endogene. The standard curve used in the M gene dataset was constructed from ACL cycle 20. The standard curves used in MYCN and Lectin datasets were from ACL cycles 27 and 29, respectively.

ACL conversions of F_N to template concentration ($C_{RNA/DNA,\ ACL}$) for the IAV M gene RNA (S11A Fig, green dots), human MYCN DNA (S11A Fig, blue squares), and soybean Lectin endogene DNA (S11A Fig, red circles) datasets were plotted as a linear regression against the known template concentrations of each dilution ($C_{RNA/DNA,\ expected}$). For each dilution, there were 3, 94, or 18 technical replicates for the M gene, MYCN, or soybean datasets, respectively. There was a clear linear relationship between $C_{RNA/DNA,\ ACL}$ and $C_{RNA/DNA,\ expected}$ for all datasets (S11A Fig, $R^2 > 0.9640$). Only 2 of the 30 total $C_{RNA/DNA,\ ACL}$ values (from all datasets) fell outside a 2-fold change (S11A Fig, black dotted lines) from $C_{RNA/DNA,\ expected}$. Of the 28 $C_{RNA/DNA,\ ACL}$ values (93% of the data) that fell within a 2-fold change, there was a maximum fold change of 1.7.

To further demonstrate the improved fit of reference amplification curves with the SCF-E model (Eq. S5) as opposed to the SCF model [3] [4], the M gene dataset was also converted using a reference curve fit with the SCF model (S11A Fig, gray dots). There was a weaker linear relationship between $C_{RNA,\ ACL}$ and

$C_{RNA, expected}$ (R^2 = 0.8487), with 8 of the 21 $C_{RNA/DNA, ACL}$ values falling outside of the 2-fold from $C_{RNA, expected}$. Furthermore, we compared ACL standard curves to *Ct* standard curves for all three template sequences. Conversion with *Ct* standard curves showed a similar linear relationship between $C_{RNA/DNA, ACL}$ and $C_{RNA/DNA, expected}$ for all datasets (S11B Fig, $R^2 > 0.9912$). This time, all measured $C_{RNA/DNA, ACL}$ values fell within a 2-fold change of the expected mean with a maximum fold change of 1.9.

Results

1. Ruijter JM, Pfaffl MW, Zhao S, Spiess AN, Boggy G, Blom J, et al. Evaluation of qPCR curve analysis methods for reliable biomarker discovery: bias, resolution, precision, and implications. Methods. 2013 Jan;59(1):32–46.

2. Lievens A, Van Aelst S, Van den Bulcke M, Goetghebeur E. Enhanced analysis of real-time PCR data by using a variable efficiency model: FPK-PCR. Nucleic Acids Res. 2012 Jan;40(2):e10.

3. Rutledge RG. Sigmoidal curve-fitting redefines quantitative real-time PCR with the prospective of developing automated high-throughput applications. Nucleic Acids Res. 2004 Dec;32(22):e178.

4. Rutledge RG, Stewart D. A kinetic-based sigmoidal model for the polymerase chain reaction and its application to high-capacity absolute quantitative real-time PCR. BMC Biotechnol. 2008 May;8:47.
