# Supplementary material for: Influenza A viral burst size from thousands of infected single cells using droplet quantitative PCR (dqPCR)
Source: PLoS Pathog. 2024 Jul 1;20(7):e1012257. doi: 10.1371/journal.ppat.1012257 (PMC11244780; doi:10.1371/journal.ppat.1012257)
Supplement: S6 Results — (PDF) [file ppat.1012257.s015.pdf]

(S6 Results) Validating dqPCR for Multiple PCR Cycle Numbers. To examine the contribution of error within individual PCR cycle numbers to overall error in the dqPCR distributions (Fig 3B), we performed an Analysis of Variance (ANOVA) test (S15 Fig) on random samples of 100 drops from each M gene distribution in Fig 3B. For the 17.1 M gene cpd group (sampled at N = 22 to 25), measured cpd differed by cycle number (p-value <0.05) and accounted for 8% of the total error in pooled measurements. For the 171 M gene cpd group (sampled at N = 19 to 22), measured cpd differed by cycle number (p-value <0.05) and accounted for 59% of the total error in pooled measurements. For the 1710 M gene cpd group (sampled at N = 6 to 22), M gene measured cpd differed by cycle number (p-value <0.05) and accounted for 7% of the total error in pooled measurements. The higher error in the 171 cpd was an outlier, perhaps due to experimental variability from cycle 19, as error was reduced to 18% when that cycle was excluded; nevertheless, the other concentrations showed relatively lower error. To address this concern, we further examined the reported variability between cycle numbers using another metric, the linear mixed effects model (LME, S16 Fig). The percent difference (%) (Eq. S14) between the measured and expected M gene cpd was calculated at each sampled cycle number. The LME used cycle number as the fixed effect variable and expected M gene cpd as the random effect variable. Both variables were log-transformed during the model fit, as replication cycles correspond to RNA copy numbers on a log scale. The fixed effects coefficients described a negative relationship between the slope and percent difference (%) from the expected M gene cpd of -0.85% per cycle number (p-value <0.05, SE = 0.01). While the p-value indicates significance, the magnitude of the relationship between cycle and converted cpd is small (~1%), reinforcing the validity of pooling data converted at different cycle numbers together. Furthermore, when we pool cycle numbers in our control experiment (Fig 3B), distributions were centered around the expected mean (S6 Table) and had a linear relationship between measured and expected cpd, that also fell within a 2-fold change from the expected mean (S17 Fig).
