# Supplementary material for: Influenza A viral burst size from thousands of infected single cells using droplet quantitative PCR (dqPCR)
Source: PLoS Pathog. 2024 Jul 1;20(7):e1012257. doi: 10.1371/journal.ppat.1012257 (PMC11244780; doi:10.1371/journal.ppat.1012257)
Supplement: S7 Results — (PDF) [file ppat.1012257.s016.pdf]

(S7 Results) Extracting Individual Distributions from Mixed Droplet Detection Data. To use dqPCR for measuring IAV burst size across a population of single-cell infections, which we expected to be heterogeneous, we validated that our method allows for isolation of individual distributions from a sample containing multiple M gene RNA concentrations. This was done by applying a Gaussian mixture model (GMM) (S7 Table) to the distributions shown in Fig 3E.

We began by fitting three log-normal distributions to the three individual concentrations of M gene cpd that make up the mixed drop distribution in Fig 3E. We let x_i be M gene cpd such that, at x_1 = 1.71 × 10^1, x_2 = 1.71 × 10^2, or x_3 = 1.71 × 10^3 cpd. The dqPCR model attributes measurement y_i to each drop. Because of measurement noise, x_i and y_i are generally not equal. Mathematically, we represent the relationship between the measurement y_i of a drop containing x_i cpd, using Eq. S15:

$$\mathbf{log_{10}}(\boldsymbol{y_i}) = \mathbf{log_{10}}(\boldsymbol{x_i}) + \boldsymbol{E_i}$$

(Eq. S15)

Here, E_i is the measurement noise. Inspired by the shape of the distribution in Fig 3E, we assumed that E_i follows a Gaussian distribution with mean B_i (representing measurement bias) and variance σ_i^2 (representing the amount of measurement noise). Each Gaussian has a different weight corresponding to the probability that a random drop in the mixture has x_i copies. Hence, to fit data in Fig 3E, we needed to estimate these weights w_i along with the B_i and σ_i^2 values. The maximum likelihood estimates are represented in S7 Table. In general, our model describes the measured distribution well, as seen from the fit in Fig 3E.

Results from S7 Table suggest that the measurement bias $\widehat{B_i}$ decreases linearly with increases in the cpd on a log scale. We therefore used a linear function to describe the relationship in Eq. S16:

$$\boldsymbol{B}(\boldsymbol{x}) \approx -\mathbf{0.06} \times \mathbf{log_{10}}(\boldsymbol{x}) + \mathbf{0.20}$$

(Eq. S16)

Here, the constants in Eq. S16 are estimated from the values reported in S7 Table.

For the standard deviation of the measurement noise, $\widehat{\sigma_i}$, we notice that the change is non-monotonic with respect to increases in cpd. We therefore fitted a quadratic curve to describe their relationship in Eq. S17:

$$\boldsymbol{\sigma(x) = 0.63 - 0.47x + 0.11x^2}$$

(Eq. S17)

Here, the constants are also estimated from the values reported in S7 Table.
