# Supplementary material for: Influenza A viral burst size from thousands of infected single cells using droplet quantitative PCR (dqPCR)
Source: PLoS Pathog. 2024 Jul 1;20(7):e1012257. doi: 10.1371/journal.ppat.1012257 (PMC11244780; doi:10.1371/journal.ppat.1012257)
Supplement: S8 Results — (PDF) [file ppat.1012257.s017.pdf]

(S8 Results) Filtering of Drops Containing Cell Lysate from Burst Size Distributions. To ensure that burst size measurements are solely from extracellular viral particles, and do not include intracellular viral RNA from cell lysate, we developed a multiplexed dqPCR assay that simultaneously detects cellular β-actin mRNA and IAV M gene RNA. β-actin was chosen as a cellular indicator due to its abundance as a structural protein, its highly conserved sequence between cell types, and its linear relationship to cell concentration (S18 Fig). For each burst size replicate experiment (S8 Table), two reference amplification curves were generated using 10^2 cpd of M gene and β-actin template controls (S1 Table) in 50 μm drops. As expected, drops containing high β-actin mRNA also exhibited high concentrations of M gene RNA (S19 Fig). These drops were presumed to contain non-packaged, intracellular M gene RNA. To remove these drops from the burst size data, we used one of two thresholds. The threshold was set at either the top 12.5% of drops containing β-actin, corresponding to the microfluidic chip split ratio, or above a β-actin concentration of 2×10^3 cpd, corresponding to the amount of β-actin released from a lysed cell (slope of Figure S18, ≈2×10^3 β-actin copies per cell). The threshold which removed the most drops was chosen to ensure that drops containing lysed cells were excluded from the data.
