# Supplementary material for: Influenza A viral burst size from thousands of infected single cells using droplet quantitative PCR (dqPCR)
Source: PLoS Pathog. 2024 Jul 1;20(7):e1012257. doi: 10.1371/journal.ppat.1012257 (PMC11244780; doi:10.1371/journal.ppat.1012257)
Supplement: S9 Results — (PDF) [file ppat.1012257.s018.pdf]

(S9 Results) Oseltamivir (OST) treatment of IAV infected cells during drop infections. To demonstrate that burst size measurements include only RNA from extracellular viral particles, we measured IAV production from single cells treated with oseltamivir acid (OST), the active metabolite of the antiviral drug oseltamivir. OST functions as a neuraminidase inhibitor, preventing the cleavage of assembled IAV particles from the host cell membrane. MDCK cells, a commonly used cell line for producing high-titer viral stocks, were infected with IAV H1N1 were encapsulated into 100 µm diameter microfluidic drops using methods for 'IAV Infection' and 'Droplet Encapsulation of Infected Cells' described in the main text. During encapsulation, infected cells were either suspended in standard droplet infection media (+H1N1 -OST) or in droplet infection media treated with 10 µM concentration of oseltamivir (+H1N1 +OST). Mock infected cells encapsulated in standard infection media (-H1N1 -OST) were used as a negative control. M gene abundance (copies/µL) was measured using a bulk RT-qPCR assay from the supernatant of broken drops sampled at 0 and 18 hpi. The 0 hpi bulk samples were frozen overnight at -80 °C while the 18 hpi bulk samples were incubated overnight at 37 °C. Preparation of drop infections for bulk RT-qPCR involved freezing a 400 µL sample of drops at -80 °C for 30 mins to break the emulsion. 300 µL of the broken emulsion was collected for further processing. Collected bulk and drop infections were clarified by centrifugation at 500 × *g* for 5 min, and the resulting supernatant was sampled for RT-qPCR targeting the IAV M gene. IAV production in drops had a mean of 1.930×10^3 M gene copies/µl at 18 hpi, across three technical replicates (S21 Fig). In the presence of oseltamivir**,** IAV production was detected in only one of three technical replicates, and measured 4.004 copies/µl. This was a significant decrease according to a two-sample Student's t-test (p <<0.05). These results support that burst size measurements include only IAV particles released from the host cell membrane and are not contaminated by exosomal RNA or other forms of extracellular contamination, as oseltamivir prevents viral budding and release.
