# Supplementary material for: Influenza A viral burst size from thousands of infected single cells using droplet quantitative PCR (dqPCR)
Source: PLoS Pathog. 2024 Jul 1;20(7):e1012257. doi: 10.1371/journal.ppat.1012257 (PMC11244780; doi:10.1371/journal.ppat.1012257)
Supplement: S10 Results — (PDF) [file ppat.1012257.s019.pdf]

(S10 Results) Modeling IAV Burst Size Distributions from Droplet RNA Concentration. The measured burst sizes distributions in Fig 4B are compounded by two underlying distributions, the viral burst size distribution and the measurement noise. To infer the viral burst size distribution, we first estimated the measurement noise by fitting log-normal distributions to fluorescence data for a mixed sample of three known M gene RNA concentrations in drops (Fig 3E, 1.71×10^1, 1.71×10^2, or 1.71×10^3 cpd). We found that the standard deviation of the distributions ranged from 0.15 - 0.30 on a $\log_{10}$ scale (S7 Table) and used this to describe the measurement noise.

We then fit a model incorporating the measurement noise and three parametric distributions, Poisson, negative-binomial and log-normal (S22 Fig), against the burst size measurements in Fig 4B. First, we simulated the viral burst size from the assumed distribution. For each simulated value of burst size x, we generated simulated measurement noise assuming a log-normal distribution with a mean defined by the bias function $B(x)$ and a standard deviation defined by $\sigma(x)$. Then, we computed a density function (kernel density with a Gaussian kernel) for the resulting distribution and used this function to compute the log-likelihood of observations. We estimated the parameters of each distribution and for each of the H3N2 or H1N1 populations by maximizing the likelihood of observations reported in Fig 4B. To reduce the computational cost, we first obtained rough parameter estimates by simulating 10,000 values from each potential burst size distribution. The ensuing estimates were then refined by repeating the above procedure using 100,000 values while constraining parameter values to a domain closer to the rough estimates.

Comparing the goodness-of-fit of the three modeled distributions to the burst size data using Akaike Information Criterion (AIC) scores, we determined that the burst size measurements were best described by a negative-binomial distribution (S10 Table and Fig 4D). The estimated mean burst sizes of these negative binomial distributions were 709 and 358 for H3N2 and H1N1, respectively. The shape parameter of each negative binomial distribution was estimated to be 0.48 and 0.49 for H3N2 and H1N1, respectively. Note that the negative-binomial distribution is considered highly dispersed when the shape

parameter is less than 1. Therefore, there exists large heterogeneity in the burst size distribution for both H1N1 and H3N2.
