# Supplementary figures and images for: Influenza A viral burst size from thousands of infected single cells using droplet quantitative PCR (dqPCR)
Source: PLoS Pathog. 2024 Jul 1;20(7):e1012257. doi: 10.1371/journal.ppat.1012257 (PMC11244780; doi:10.1371/journal.ppat.1012257)

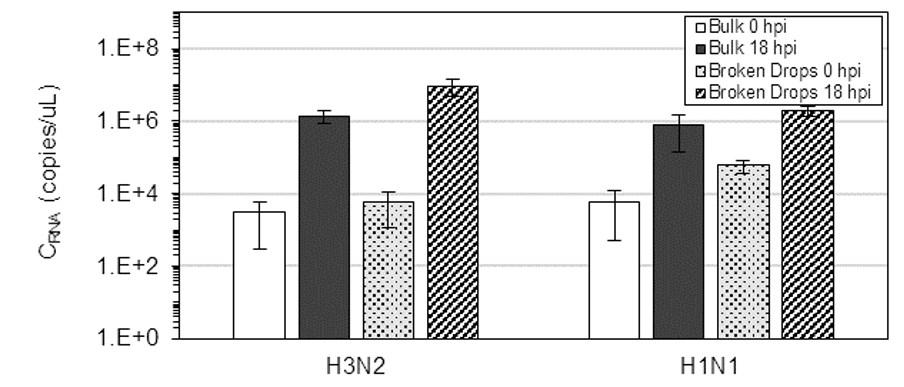

Supplement: S1 Fig — IAV strains (H1N1 and H3N2) were separately used to infect A549 cells under bulk cell culture and single cell encapsulation in 100 μm diameter microfluidic drops. M gene abundance (copies/μL) was measured using a bulk RT-qPCR assay from the supernatant of both bulk and drop infections at 0 and 18 hpi. Experimental details can be found in S1 Results. Each bar represents the pooled data from three replicate experiments. Error bars represent one standard deviation. (TIF) [file ppat.1012257.s020.tif]

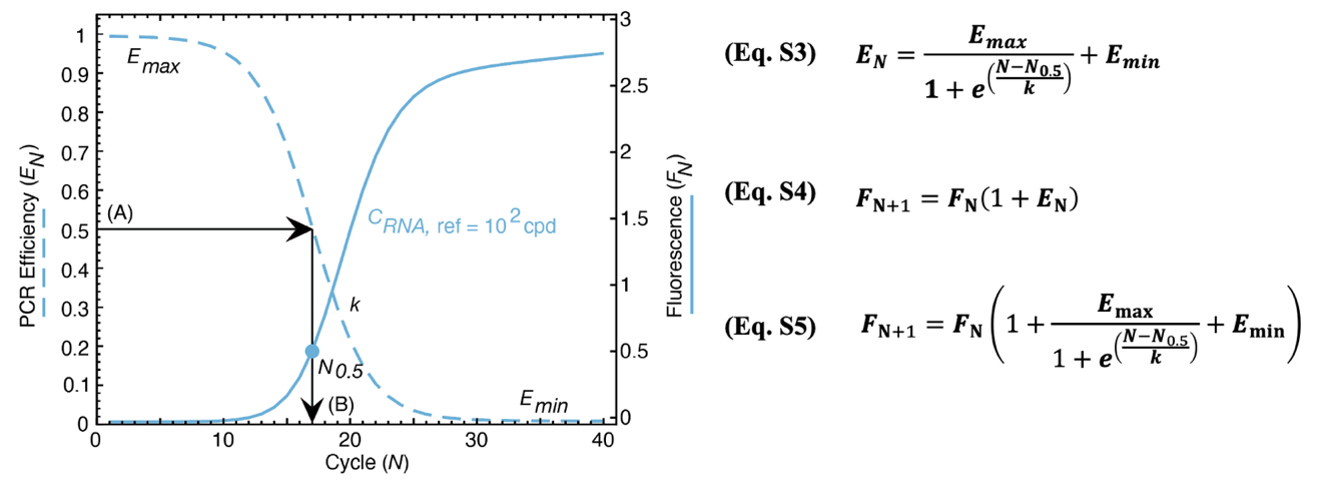

Supplement: S2 Fig — A four-parametric sigmoid function was used to model decreasing PCR efficiency (EN) at each cycle number (N) (Eq. S3). This model was fitted to discontinuous fluorescence measurements (FN) of a known RNA template concentration (CRNA, ref) to generate a continuous reference amplification curve (Eq. S4 and S5). Reference curves used in burst size experiments were generated with CRNA, ref = 1.71 × 10^2^ RNA (cpd). In the SCF-E model, EN is the PCR efficiency at cycle N; Emax is the maximum PCR efficiency at cycle number 1 and ranges from of 0.9 to 1; Emin is the minimum PCR efficiency at cycle number 40 and ranges from 0 to 0.1; N0.5 is the cycle number at which EN equals 0.5; and k is the shape parameter of the curve. As k increases, the shape of the curve flattens, and as k decreases, the shape of the curve becomes steeper. Thirty values of each of the four efficiency parameters (Emax, Emin, N0.5, and k) were used to test 8.1×10^5^ (304) curve fits to experimental fluorescence measurements. The set of efficiency parameters yielding the best fit were used to construct the SCF-E reference curve (solid blue curve). (TIF) [file ppat.1012257.s021.tif]

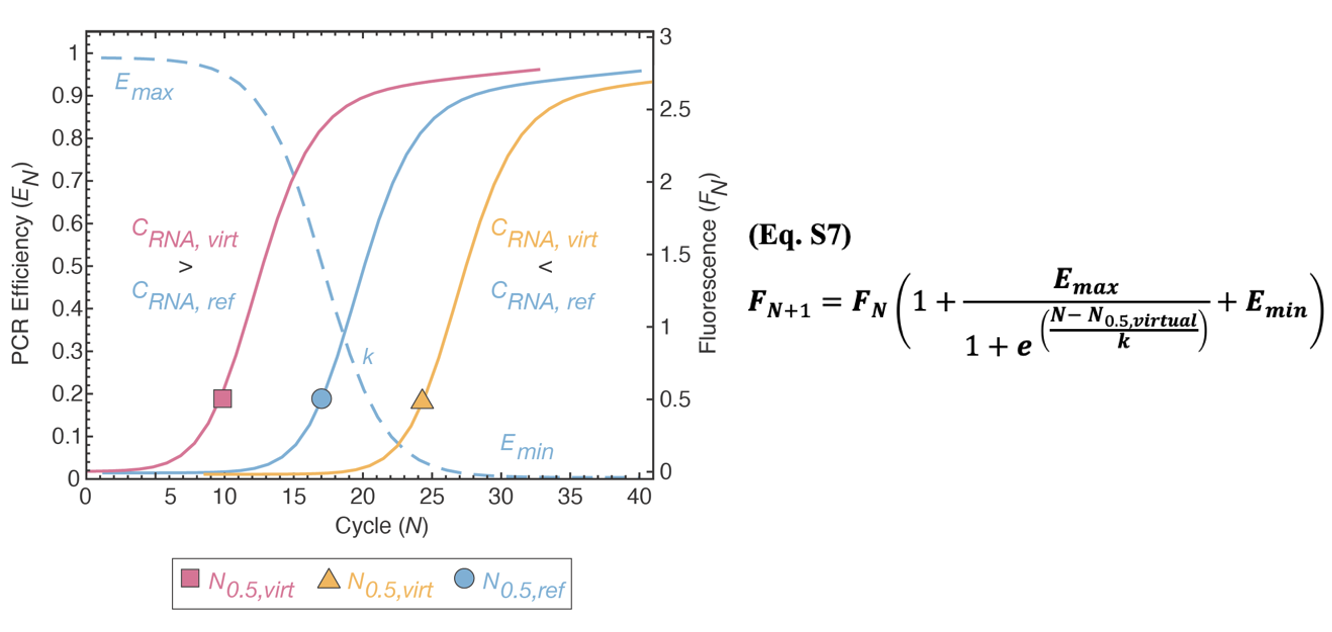

Supplement: S3 Fig — ACLs consist of virtual amplification curves that share the same Emax, Emin, and k parameters as the reference curve but have unique N0.5 values. This is done by substituting N0.5, ref from Eq. S5 with N0.5, virt in Eq. S7. We input 1000 evenly spaced N0.5, virt values between cycle numbers N = 1 to 40, resulting in 1000 virtual amplification curves, each associated with a unique theoretical RNA concentration (CRNA, virt). Virtual curves with N0.5, virt < N0.5, ref (leftmost pink solid curve) correspond to higher RNA concentrations compared to the reference curve, whereas curves with N0.5, virt > N0.5, ref (rightmost yellow solid curve) represent lower RNA concentrations than the reference (middle blue solid curve). The efficiency curve for reference and virtual curves is illustrated as a dashed blue line. (TIF) [file ppat.1012257.s022.tif]

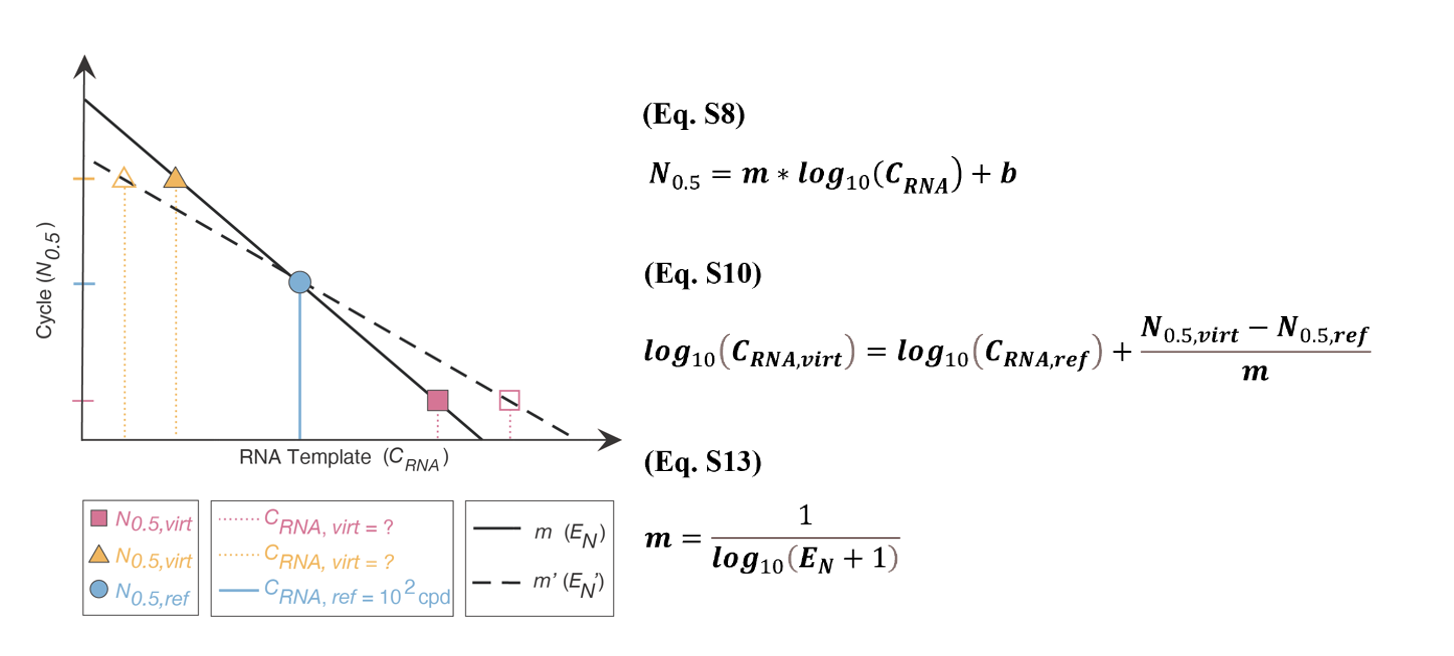

Supplement: S4 Fig — We relate the known RNA concentration of the reference curve (CRNA, ref) to the unknown RNA concentrations of the virtual curves (CRNA, virt) based on their position on the x-axis (cycle number, N). In Eq. S10, we define a relationship between the known cycle numbers where PCR efficiency is equal to 0.5 for the reference (N0.5, ref) and virtual (N0.5, virt) curves and their respective RNA concentrations (CRNA). By rearranging Eq. S10, we can calculate the unknown CRNA, virt using Eq. S12. To perform the calculation, the slope of the line (m) in Eq. S13 is determined using a PCR efficiency constant (EN). As indicated by Eq. S13, an increase in EN leads to a decrease in m (dotted black line), expanding the range of CRNA, virt in the ACL (from solid triangle to open triangle, and from solid square to open square). This relationship is visually illustrated here with Eq. S8. (TIF) [file ppat.1012257.s023.tif]

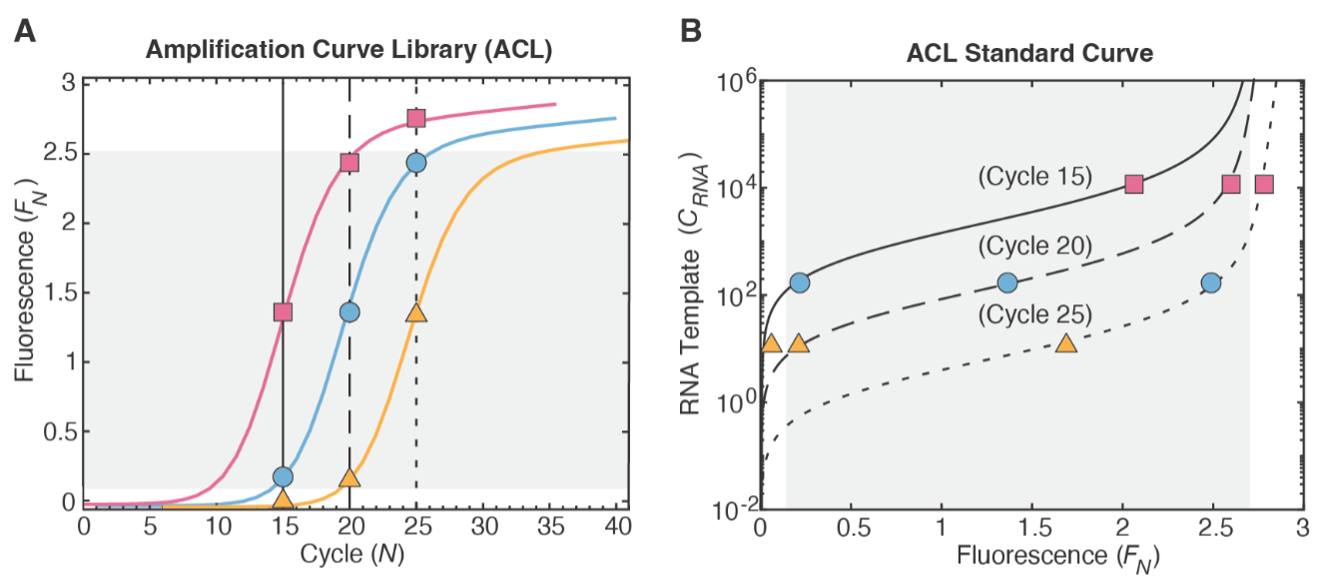

Supplement: S5 Fig — (A) Construction of an amplification curve library (ACL) (S3 Fig). Three CRNA, virt within the ACL are displayed here (pink, blue, yellow), with the leftmost pink curve representing a higher CRNA, virt compared to the rightmost yellow curve. (B) ACL standard curves (solid line, dashed line, dotted line) are constructed from the FN of CRNA, virt (pink, blue, yellow) at a particular cycle number (15, 20, 25). The cycle number chosen for the ACL standard curve corresponds to the cycle number at which unknown RNA concentrations are sampled. In this method, only virtual curves in the exponential or linear phase at the selected cycle number can be included in the standard curve (grey shaded regions in A and B). This is because the early amplification and plateau regions of multiple virtual curves may overlap at a single cycle number. To ensure that only useable regions are selected for the standard curve, we establish threshold values based on the fluorescence of the reference curve. The upper threshold is set at 1 standard deviation below the FN at cycle 40, and the lower threshold is set at the 99th percentile of the FN at cycle 1. For example, as amplification proceeds for a particular CRNA, virt (pink curve) in A, the FN increases and intersects cycles 15, 20, and 25 at the three points (squares). However, only two of those points are within the valid range of the ACL standard curves in B. (TIF) [file ppat.1012257.s024.tif]

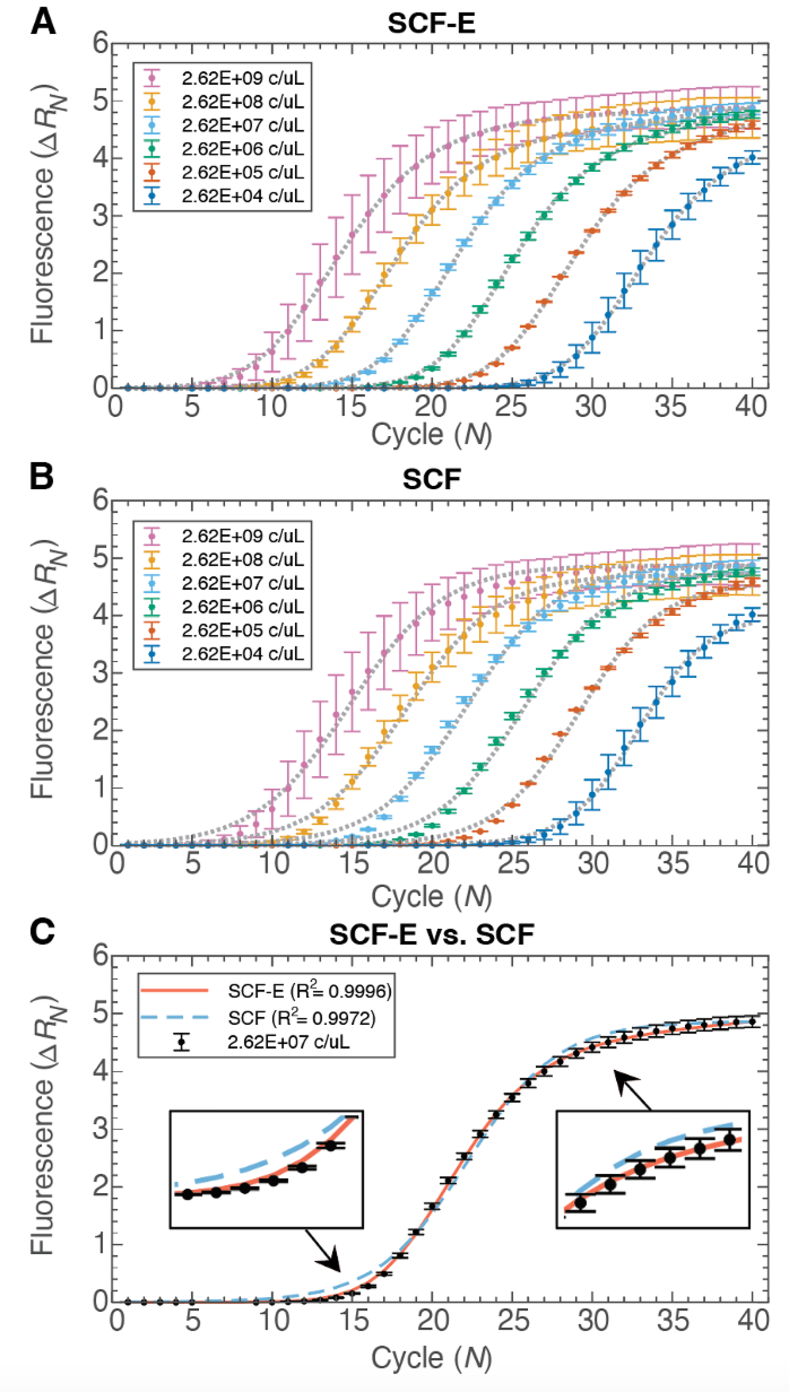

Supplement: S6 Fig — Fluorescence intensities (ΔRN) of bulk RT-qPCR amplification of six concentrations of IAV M gene RNA (S2 Table, 10-fold dilutions from 2.62 × 10^4^ to 2.62 × 10^9^ copies/μL) were measured at N = 1 to 40. The concentration of each dilution decreases from left to right when viewing the amplification curves. (A) Amplification curves generated with the SCF-E model (dotted gray curves) had an average fit of R2 = 0.9996 across all RNA concentrations (S3 Table). (B) Amplification curves generated with the SCF model (dotted gray curves) had an average fit of R2 = 0.9975 across all RNA concentrations (S3 Table). (C) For a single M gene RNA concentration (2.62 × 10^7^ copies/μL), the SCF-E model provided a better fit (R2 = 0.9996) than SCF (R2 = 0.9972). The difference in fit is most noticeable at the lower (ΔRN < 1) and higher (ΔRN > 4) fluorescence values, representing the exponential and plateau phases of PCR amplification (magnified insets). All error bars represent one standard deviation from the average fluorescence measured across three technical replicates. (TIF) [file ppat.1012257.s025.tif]

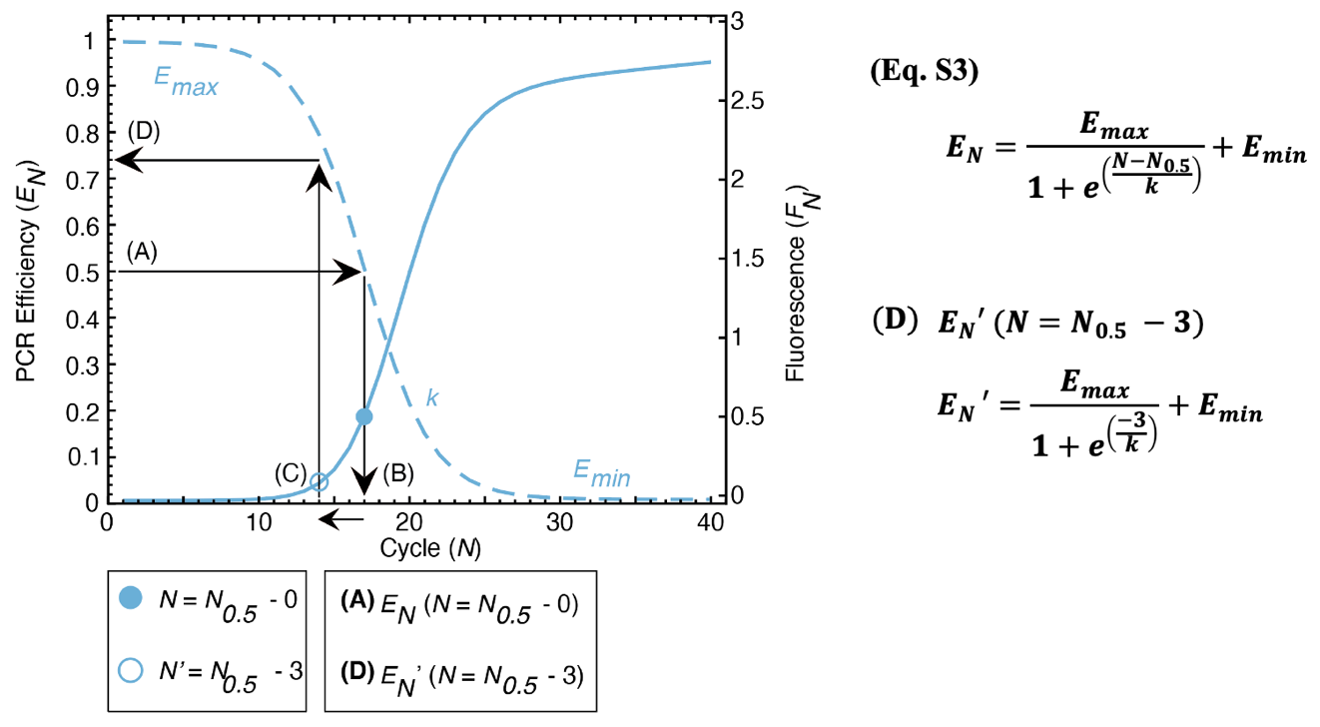

Supplement: S7 Fig — To calculate EN at N = N0.5−3, we determined N0.5 of the SCF-E reference curve, which corresponds to N where EN = 0.5 (A to B). N0.5−3 represents three cycles prior to N0.5 (C). We recorded the EN at N0.5−3 (D). When N = N0.5−3 is inserted into Eq. S3, the term N0.5 cancels out, resulting in a constant value of -3 for (N–N0.5). The EN calculated in this step is used in Eq. S13 to determine m. (TIF) [file ppat.1012257.s026.tif]

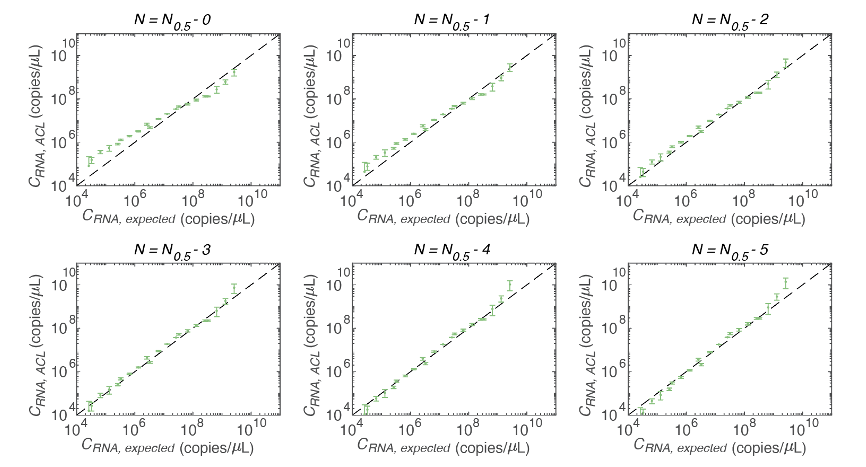

Supplement: S8 Fig — We compared cycle numbers (N) at which PCR efficiency (EN, Eq. S3) of the reference curve is used to calculate the constant m (Eq. S13), for assigning RNA template concentrations to virtual curves in the ACL. We tested the cycle number at which PCR efficiency is equal to 0.5 (N0.5) and the five cycles preceding it (N = N0.5−1 to N = N0.5−5), to construct six ACLs for comparison. From each ACL, a standard curve was constructed at PCR cycle number 20. We evaluated the dynamic range of each standard curve in S4 Table. The ACL standard curve with the widest dynamic range and the highest degree of accuracy (R2) between measured (CRNA, ACL) and expected (CRNA, expected) M gene RNA concentrations was built using EN at N = N0.5−3. For this ACL, the dynamic range of the standard curve was 4.70 log10 RNA copies/μL (S4 Table, dynamic range of 2.62 × 10^4^ to 1.31 × 10^9^ copies/μL) with R2 = 0.990 between CRNA, ACL, and CRNA, expected. Therefore, the EN at N = N0.5−3 was used to construct all amplification curve libraries in this work. (TIF) [file ppat.1012257.s027.tif]

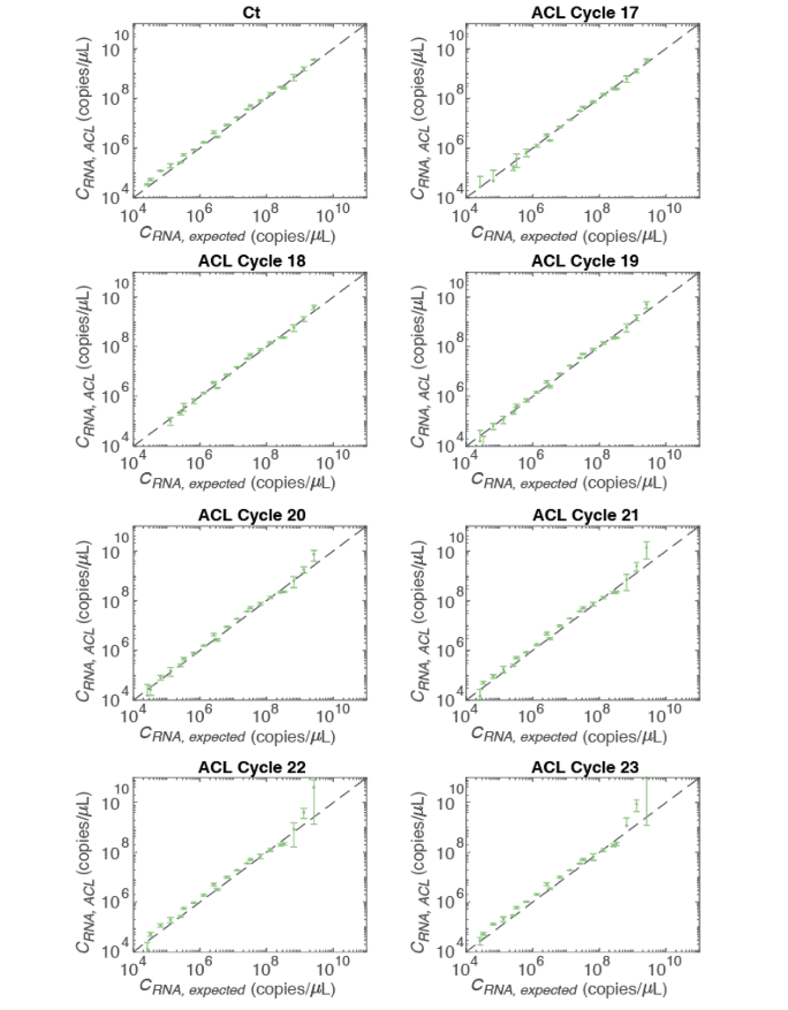

Supplement: S9 Fig — We compared the dynamic range of ACL standard curves from different cycle numbers (N = 17 to 23) to the Ct standard curve. Standard curves were used to convert fluorescence to template concentration for a known dilution series of IAV M gene RNA (S2 Table, three replicates of 21 concentrations between 2.62 × 10^4^ to 2.62 × 10^9^ copies/uL). Standard curve dynamic range and degree of accuracy (R2) between measured (CRNA, ACL) and expected (CRNA, expected) M gene RNA concentrations were evaluated in S5 Table. (TIF) [file ppat.1012257.s028.tif]

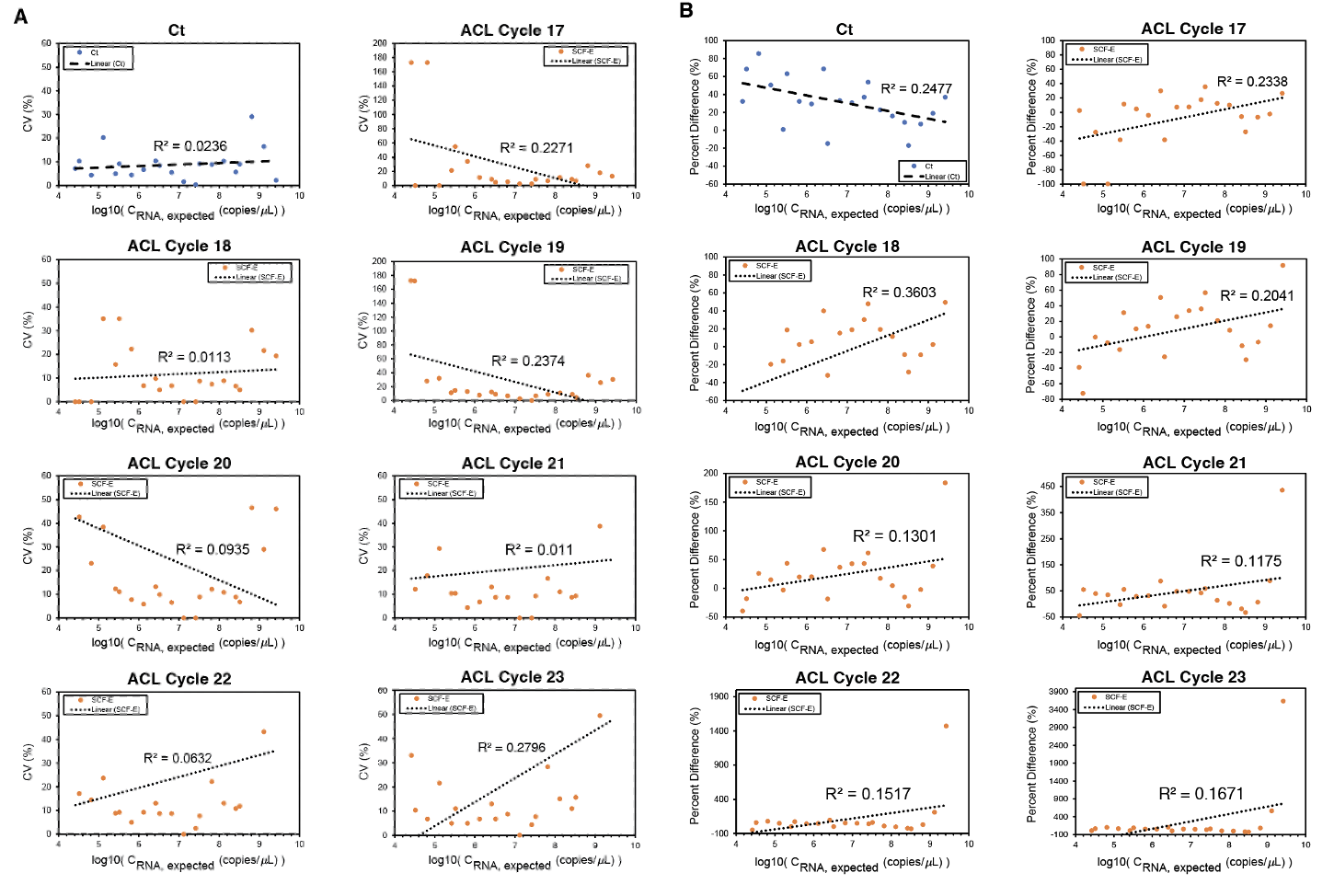

Supplement: S10 Fig — We examined variability between standard curves obtained from different ACL cycle numbers using two measures: (A) coefficient of variation (CV %) and (B) percent difference (%). Standard curves were constructed from ACL cycle numbers (N = 17 to 23, individual plots) and used to convert fluorescence to RNA concentration (CRNA, ACL) across 23 IAV M gene RNA concentrations (S2 Table). (A) CV (%) is the ratio of the standard deviation to the mean CRNA, ACL, and it quantifies the variability within replicates of the same M gene RNA concentration. A linear regression (dotted line) was conducted to compare CV (%) across M gene RNA concentrations. The results revealed a weak linear relationship (R2 < 0.28) for all cycle numbers. (B) Percent difference (%) measures the variability between CRNA, ACL and the expected RNA concentration (CRNA, expected). Similarly, a weak relationship (R2 < 0.36) was observed between the starting RNA concentration and percent difference (%) for all cycle numbers. To assess the variability in ACL standard curves, a comparison was made with Ct standard curves (A and B, first upper left plot), for CV (%) and percent difference (%). The analysis indicated no linear response for CV (%) (R2 = 0.0236) and a weak linear response for percent difference (%) (R2 = 0.2477) across the M gene RNA concentrations. (TIF) [file ppat.1012257.s029.tif]

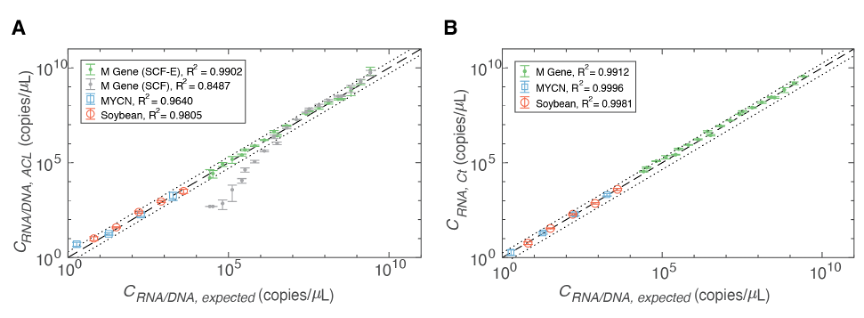

Supplement: S11 Fig — Standard curves constructed using the (A) ACL method or (B) Ct method were used to convert fluorescence to template concentration (CRNA/DNA, ACL) for a known dilution series of three different template sequences: IAV M gene RNA (green/gray dots, ranging from 2.62 × 10^4^ to 2.92 × 10^9^ copies/μL), human MYCN gene DNA (blue squares, ranging from 1.88 × 100 to 1.88 × 10^3^ copies/μL), and soybean Lectin endogene DNA (red circles, ranging from 6.4 × 100 to 4.0 × 10^3^ copies/μL). Each dilution of the templates had varying numbers of technical replicates: 3 for the M gene, 94 for the MYCN, and 18 for the Lectin dataset. The M gene ACL was built with a 107 copies/μL reference curve fitted with either the SCF-E (green dots) or SCF (grey dots) model. The standard curve from the M gene ACL was constructed at a single cycle number (N = 20). The MYCN libraries were built with a 1,875 copies/μL reference curve, and the Soybean ACL was built with an 800 copies/μL reference curve, both fitted using the SCF-E model. The standard curves from these ACLs were constructed at N = 27 (MYCN) and N = 29 (Lectin). An R2 value quantified the linear response between measured CRNA/DNA, ACL to expected concentrations (CRNA/DNA, expected) across template concentrations. Dotted lines indicate a 2-fold change from CRNA/DNA, expected, and error bars represent one standard deviation from the mean. (TIF) [file ppat.1012257.s030.tif]

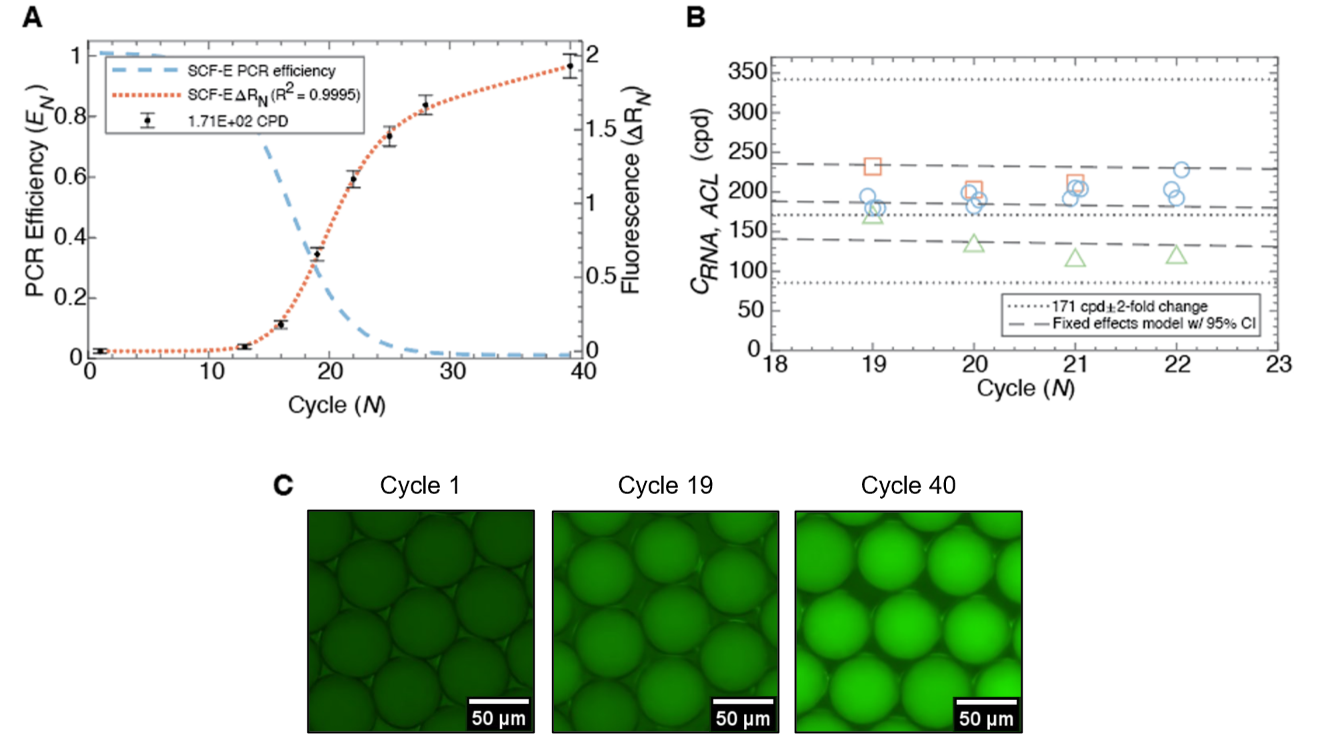

Supplement: S12 Fig — (A) SCF-E reference amplification curve generated for 1.71 × 10^2^ cpd of IAV M gene RNA amplified in 50 μm drops. Drop fluorescence intensity (ΔRN) was detected at PCR cycle numbers N = 1, 13, 16, 19, 22, 25, 28, 40 and displayed as a mean (black dots) and one standard deviation (error bars) (≈1000 or more drops per cycle number). The estimated PCR efficiency (EN) curve (dashed blue line) used during SCF-E produced a well-fitting reference amplification curve (dotted red line, R2 = 0.9995). (B) dqPCR was validated for a single concentration of M gene RNA (1.71x10^2^ cpd) across three biological replicates. The samples were collected at various cycle numbers: rep 1 (red squares) at N = 19 to 21 and rep 2 (blue circles) and rep 3 (green triangles) at N = 19 to 22. Drop fluorescence was converted to M gene RNA concentration (CRNA, ACL) within a 2-fold change of the expected mean (dotted lines) for all cycle numbers. Dashed lines indicate a fixed effects model with upper and lower bounds set as the 95% confidence intervals. (C) Representative epifluorescence images of 50 μm diameter drops at PCR cycle numbers 1, 19, and 40. (TIF) [file ppat.1012257.s031.tif]

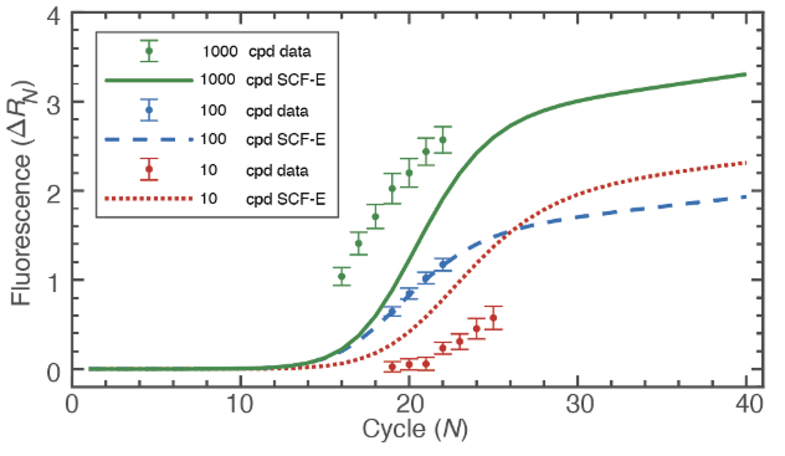

Supplement: S13 Fig — Three known concentrations of M gene RNA, 1.71 × 101 cpd (red dots), 1.71 × 10^2^ cpd (blue dots), and 1.71 × 10^3^ cpd (green dots), were amplified in 50 μm drops. Drop fluorescence (ΔRN) was detected at multiple PCR cycle numbers (S6 Table, note that cycles 19–21 in the 10 cpd group were below background and not included in the table) and converted to M gene cpd using dqPCR (Fig 3B). For each experiment run on different days, a new SCF-E reference curve was generated from the amplification of 1.71 × 10^2^ cpd (red dotted line, blue dashed line, green solid line). (TIF) [file ppat.1012257.s032.tif]

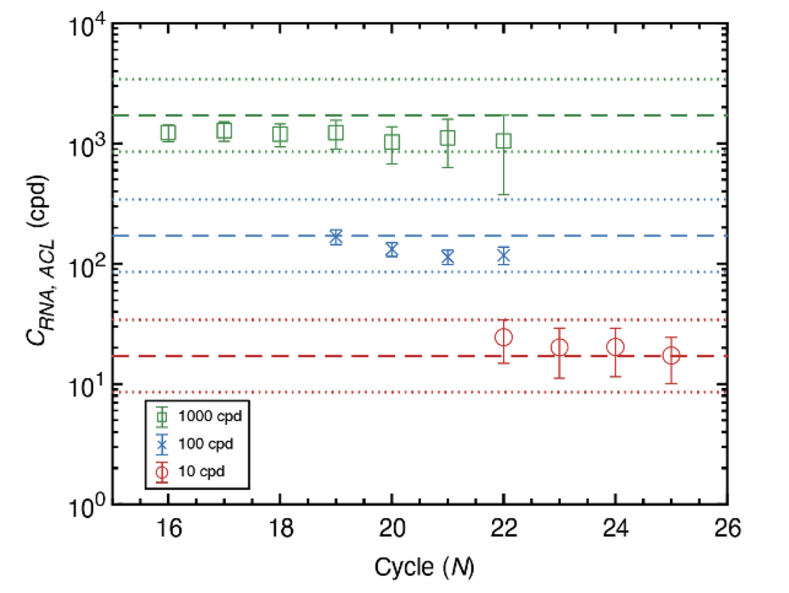

Supplement: S14 Fig — Three known IAV M gene RNA concentrations, 1.17 × 101 cpd (10 cpd, red circles), 1.17 × 10^2^ cpd (100 cpd, blue crosses), and 1.17 × 10^3^ cpd (1000 cpd, green squares) were amplified in 50 μm drops. Drop fluorescence (ΔRN) was detected at multiple PCR cycle numbers (S6 Table) and converted to M gene cpd (CRNA, ACL) using dqPCR. CRNA, ACL measurements were pooled together and displayed as a single distribution in Fig 3B. Dashed lines indicate the expected M gene cpd, and dotted lines represent a 2-fold change from the expected mean. (TIF) [file ppat.1012257.s033.tif]

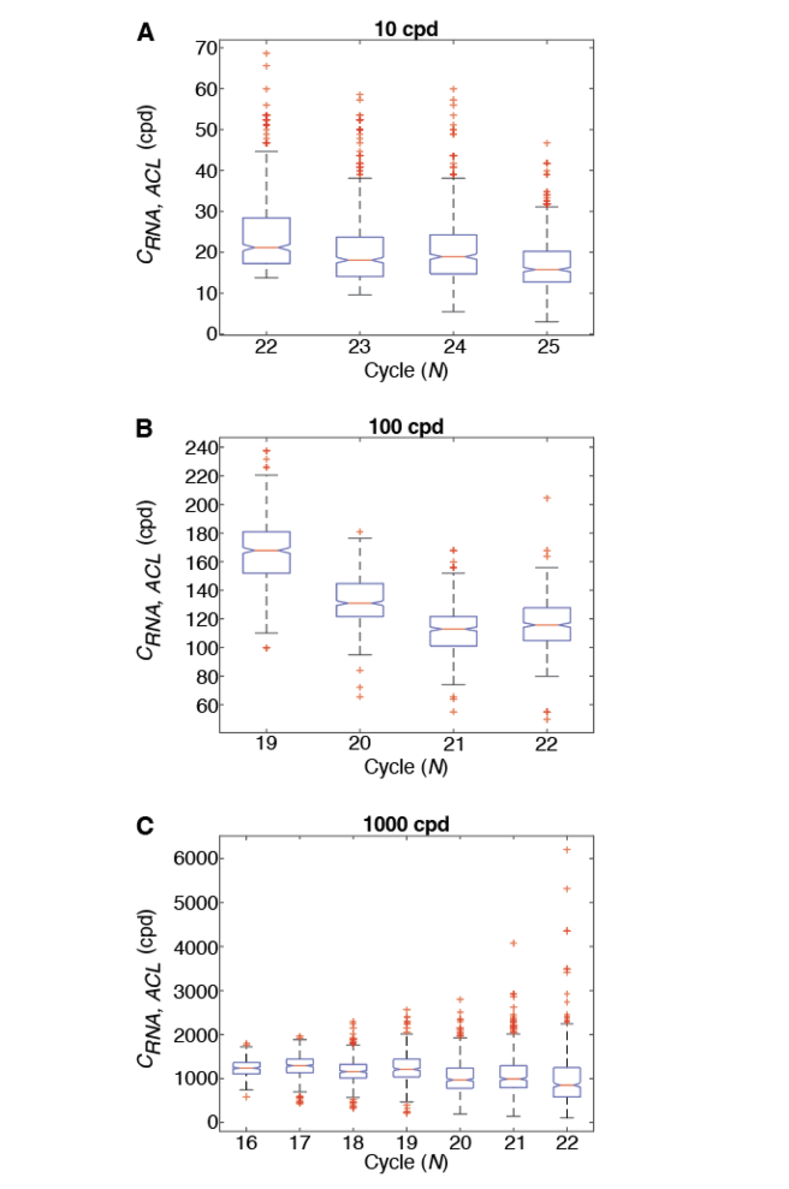

Supplement: S15 Fig — Three known IAV M gene RNA concentrations, 1.17 × 101 cpd (10 cpd plot), 1.17 × 10^2^ cpd (100 cpd plot), and 1.17 × 10^3^ cpd (1000 cpd plot), were amplified in 50 μm drops. Drop fluorescence (ΔRN) was detected at multiple PCR cycle numbers (S6 Table) and converted to M gene cpd (CRNA, ACL) using the dqPCR model. CRNA, ACL measurements were pooled together and displayed as a single distribution in Fig 3B. To examine the contribution of error within individual PCR cycle numbers to overall error in the pooled distributions, we performed an Analysis of Variance (ANOVA) test on a random sample of 100 drops from each group. (A) The 10 cpd group, quantified at PCR cycle numbers 22–25, had mean CRNA, ACL values that differed by cycle number (ANOVA p-value <0.05). This variability due to cycle number accounted for 8% of the total error in the pooled distribution. (B) The 100 cpd group, quantified at PCR cycle numbers 19–22, had mean CRNA, ACL values that differed by cycle number (ANOVA p-value <0.05). This variability due to cycle number accounted for 59% of the total error in the pooled distribution. (C) The 1000 cpd group, quantified at PCR cycle numbers 16–22, had mean CRNA, ACL values that differed by cycle number (ANOVA p-value <0.05). This variability due to cycle number accounted for 7% of the total error in the pooled distribution. (TIF) [file ppat.1012257.s034.tif]

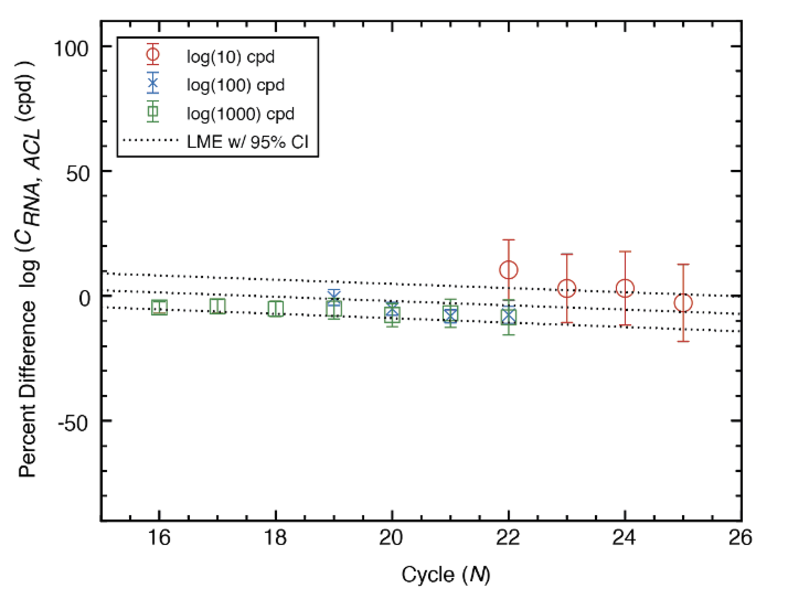

Supplement: S16 Fig — Three known IAV M gene RNA concentrations, 1.17 × 101 cpd (10 cpd, red circle), 1.17 × 10^2^ cpd (100 cpd, blue cross), and 1.17 × 10^3^ cpd (1000 cpd, green triangle), were amplified in 50 μm drops. Drop fluorescence (ΔRN) was detected at multiple PCR cycle numbers (S6 Table) and converted to M gene cpd (CRNA, ACL). CRNA, ACL measurements were pooled together and displayed as a single distribution in Fig 3B. To further examine the contribution of error within individual PCR cycle numbers to overall error in the pooled distributions, we calculated the percent difference (%) (Eq. S14) between the measured CRNA, ACL values and their expected M gene cpd (CRNA, expected), across cycle numbers. This analysis informed the validity of pooling conversions measured from multiple PCR cycles in our burst size experiments. Percent differences were compared using a linear mixed effects model (LME) with a 95% confidence interval (CI) (dotted lines). In the LME, we set cycle as the fixed effect variable and CRNA, expect as the random effect variable. Both variables were log-transformed during the model fit, as replication cycles correspond to RNA copy numbers on a log scale. The fixed effects coefficients described a negative relationship between the slope and percent difference from the expected RNA concentration of -0.85% per cycle number (p-value <0.05, SE = 0.01). While the p-value indicates significance, the magnitude of the relationship is small (~1%/cycle), maintaining that it is valid to pool dqPCR data from different cycle numbers. (TIF) [file ppat.1012257.s035.tif]

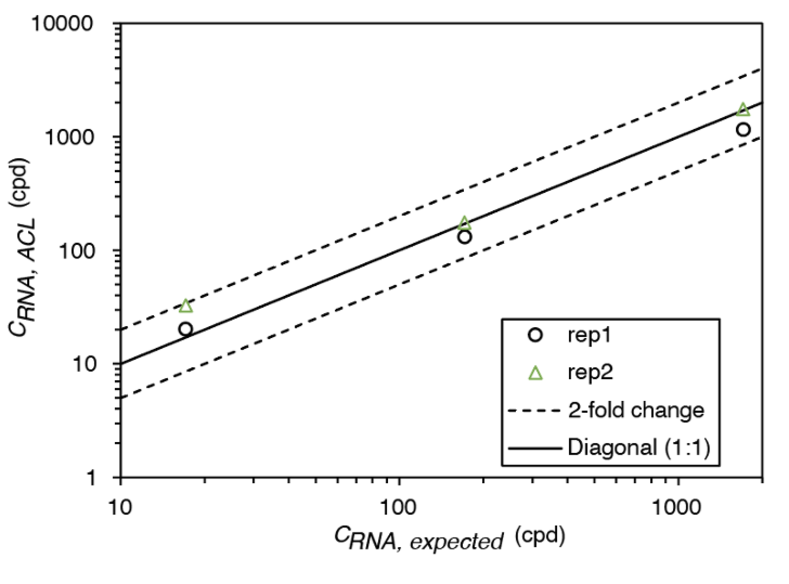

Supplement: S17 Fig — Three known IAV M gene RNA concentrations, 1.17 × 101 cpd, 1.17 × 10^2^ cpd, 1.17 × 10^3^ cpd, were amplified in 50 μm drops. Drop fluorescence (ΔRN) was detected at multiple PCR cycle numbers (S6 Table) and converted to M gene cpd (CRNA, ACL) using dqPCR. CRNA, ACL measurements were pooled together and plotted against the expected M gene cpd (CRNA, expected). Two biological replicates (black circle and green triangle) are shown. The solid line represents a 1:1 relationship between CRNA, ACL and CRNA, expected, dashed lines indicate a 2-fold change from the expected mean. (TIF) [file ppat.1012257.s036.tif]

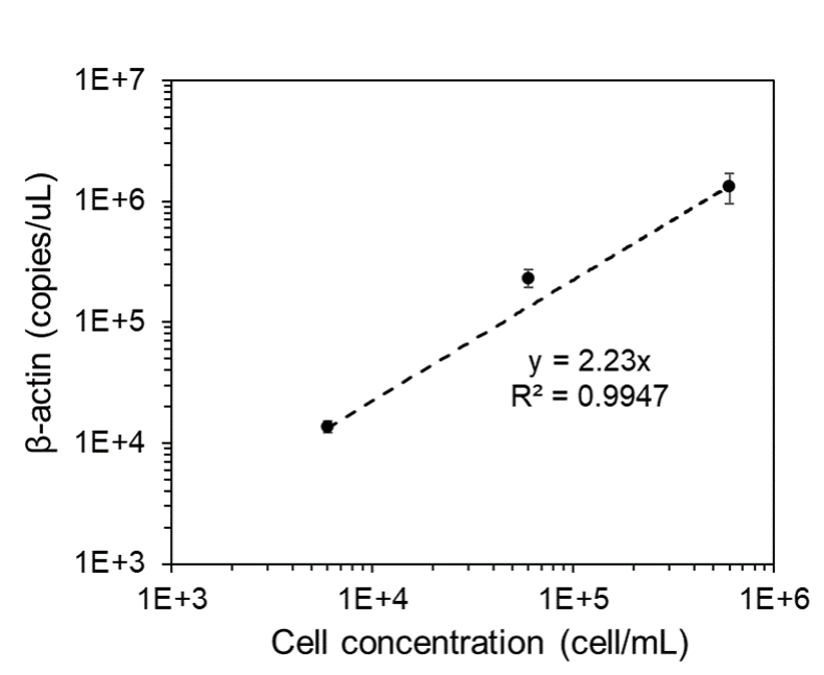

Supplement: S18 Fig — A bulk RT-qPCR assay was performed to amplify β-actin mRNA from lysed A549 cells after 24 hr of incubation. There was a strong linear relationship between β-actin and cell concentration (dashed black line, R2 = 0.995), validating its use as a reliable marker for the detection of intracellular lysate in drops during our burst size experiments. (TIF) [file ppat.1012257.s037.tif]

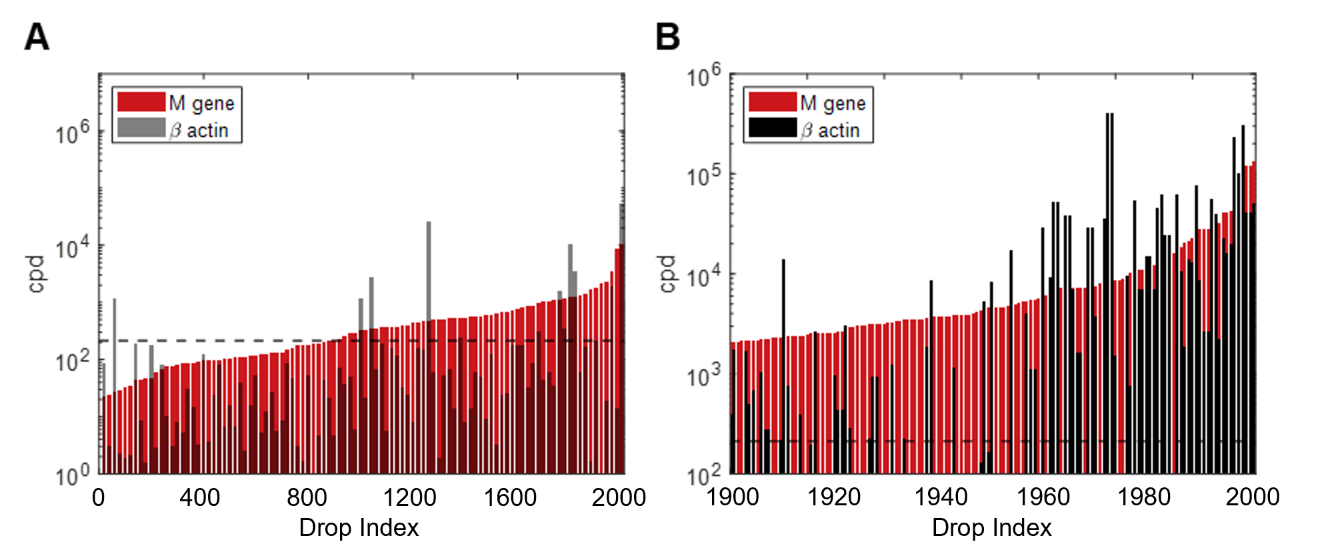

Supplement: S19 Fig — (A) Both M gene RNA (red) and β-actin mRNA (black) were detected by multiplexed dqPCR from H1N1 infection in drops (n = 2000). Data is presented in ascending order of RNA concentration, prior to β-actin filtering (indicated by the dashed black line). (B) Showing a zoomed-in region of (A), with drop indexes 1900 to 2000. (TIF) [file ppat.1012257.s038.tif]

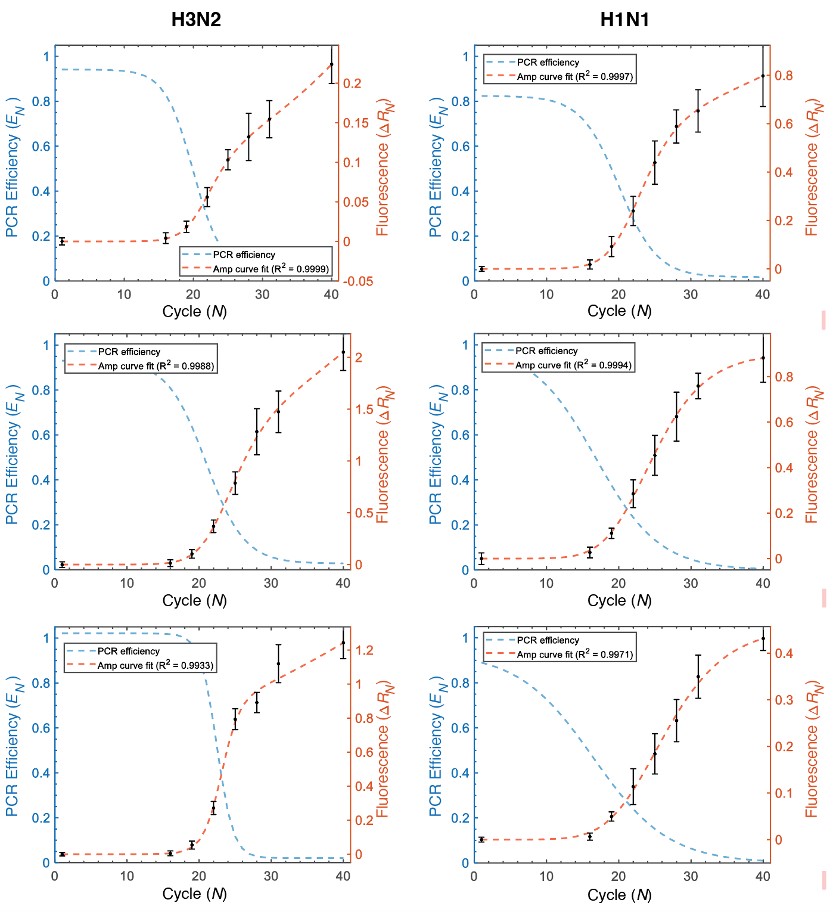

Supplement: S20 Fig — For each burst size replicate experiment (S8 Table), a new reference amplification curve was constructed for dqPCR (S9 Table). To generate these curves, a known concentration of M gene RNA (1.71 × 10^2^ cpd) was amplified in 100 μm drops. Drop fluorescence intensity (ΔRN) was measured at PCR cycle numbers N = 1, 16, 19, 22. 25, 28, 31 and 40. The ΔRN of drops from each cycle number are shown as the mean (black dots) with error bars representing one standard deviation. SCF-E was used to create a continuous reference amplification curve (orange dashed line) from discontinuous ΔRN measurements, using an estimate for the PCR efficiency (blue dashed line). (TIF) [file ppat.1012257.s039.tif]

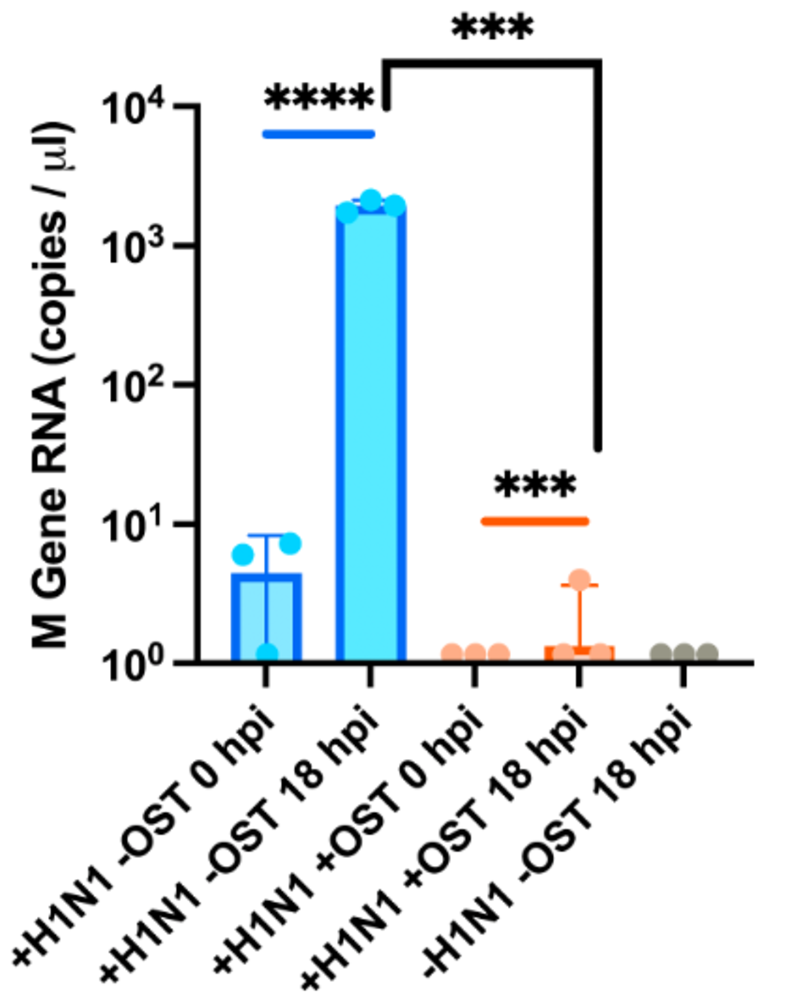

Supplement: S21 Fig — MDCK cells infected with IAV H1N1 were encapsulated into 100 μm diameter microfluidic drops. During encapsulation, infected cells were either suspended in standard droplet infection media (+H1N1 -OST) or in droplet infection media treated with 10 μM concentration of oseltamivir (+H1N1 +OST). Mock infected cells encapsulated in standard infection media (-H1N1 –OST) were used as a negative control. M gene abundance (copies / μL) was measured using a bulk RT-qPCR assay from the supernatant of broken drops sampled at 0 and 18 hpi. Each bar represents the pooled data from three technical replicates. Significance stars (*** p < 0.001 and **** p < 0.0001) obtained by a two-sample Student’s t-test. (TIF) [file ppat.1012257.s040.tif]

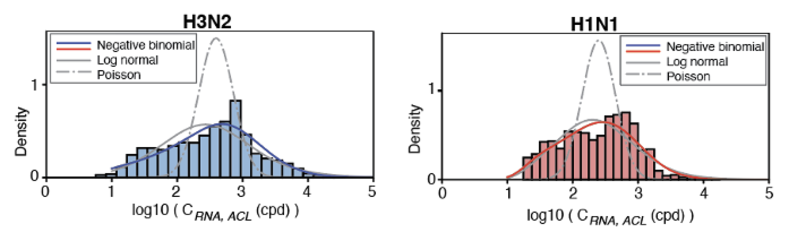

Supplement: S22 Fig — We used a simulation-based approach to estimate the IAV burst distributions shown in Fig 4D. We considered three distribution models: lognormal (solid gray curve), Poisson (dashed gray curve), and negative-binomial distribution (solid blue or red curve, corresponding to H3N2 and H1N1 distributions, respectively). The parameters for each distribution are provided in S10 Table. Based on the analysis, burst sizes were found to best fit a negative binomial distribution. (TIF) [file ppat.1012257.s041.tif]

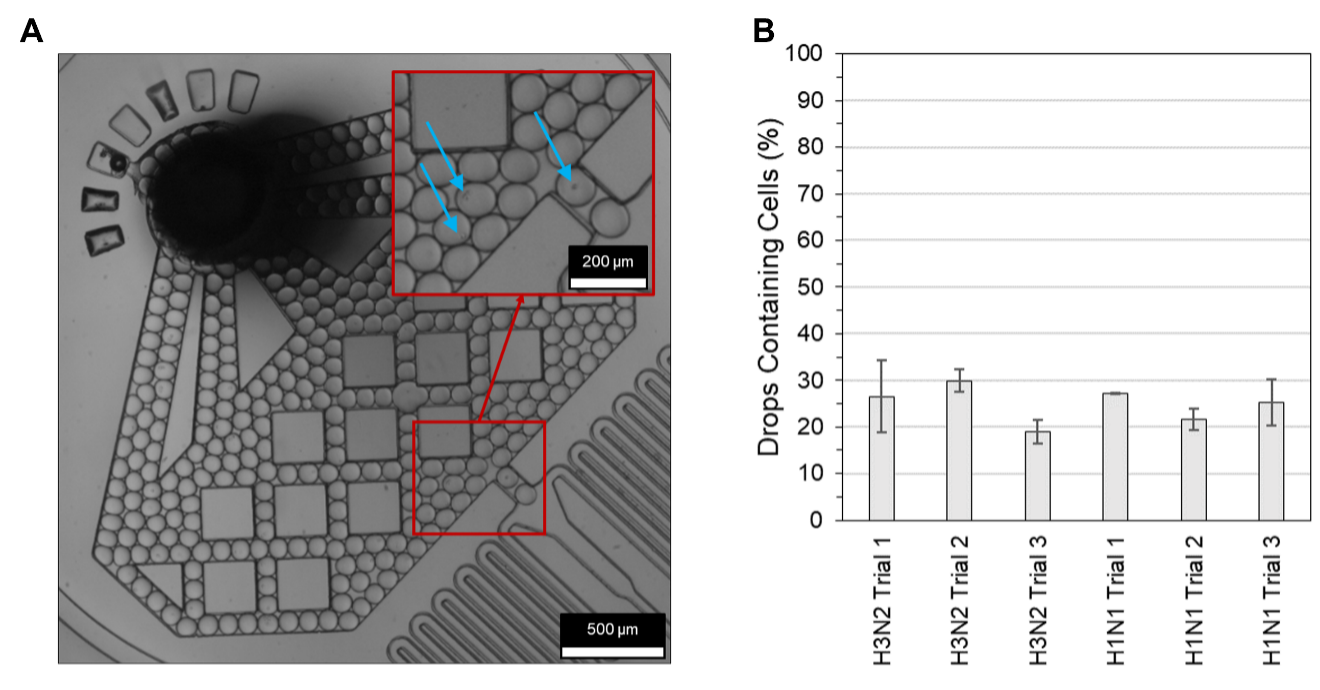

Supplement: S23 Fig — (A) Images of 100 μm drops re-injected into a microfluidic device were captured at the beginning and end of each burst size replicate experiment to monitor cell loading. Drops containing a single cell are indicated by blue arrows. (B) Across all H3N2 and H1N1 replicate experiments, cell loading was determined by counting the percentage of drops containing cells (mean = 25% ± 4%). Cell loading in drops was assumed to follow a Poisson distribution. For a population of drops with 25% containing cells, the estimated Poisson mean (λ) is 0.29 cells/drop. In this case, ≈75% of drops are empty, ≈21.5% of drops contain one cell, ≈3% contain two cells, and ≈0.5% of drops contain three or more cells. The bar graph displays the drop counts for the replicates from left to right, which are 753, 679, 538, 719, 774, and 582. (TIF) [file ppat.1012257.s042.tif]

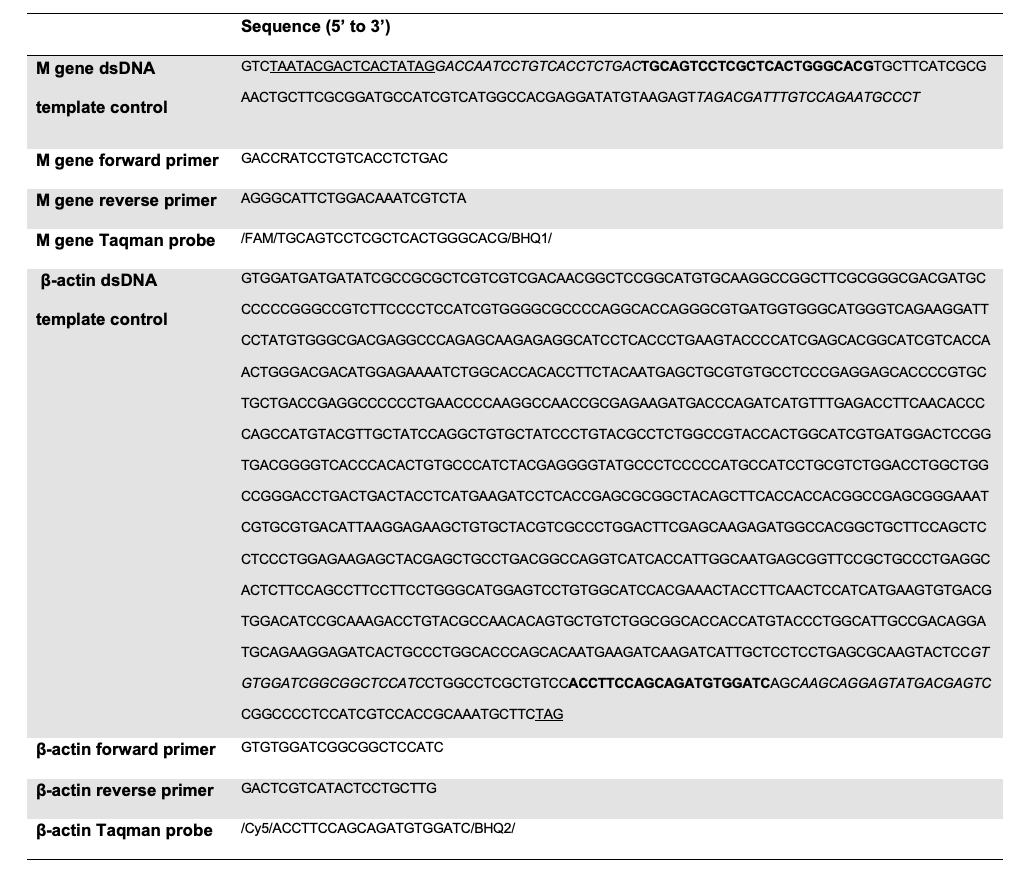

Supplement: S1 Table — All sequences are listed in the 5’ to 3’ direction. The M gene template control was constructed as linear dsDNA containing a T7 promoter (underlined), forward and reverse primer binding sites (italicized), a probe binding site (bold), and a partial M gene sequence. The M gene dsDNA was in vitro transcribed prior to use as a positive control. The M gene TaqMan probe carried a FAM fluorophore with a BHQ1 quencher. The β-actin template control was constructed as a dsDNA plasmid containing forward and reverse primer binding sites (italicized), and a probe binding site (bold), and a partial β-actin mRNA sequence. The β-actin TaqMan probe carried a Cy5 fluorophore with a BHQ2 quencher. (TIF) [file ppat.1012257.s043.tif]

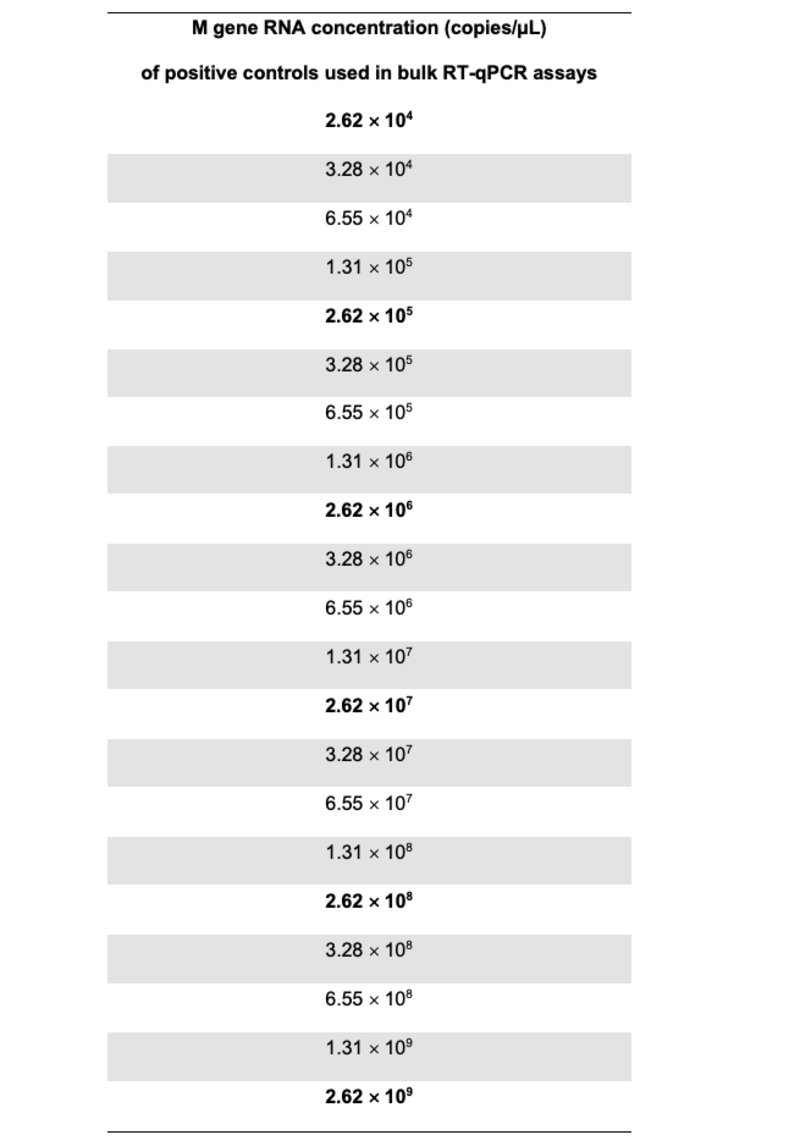

Supplement: S2 Table — (TIF) [file ppat.1012257.s044.tif]

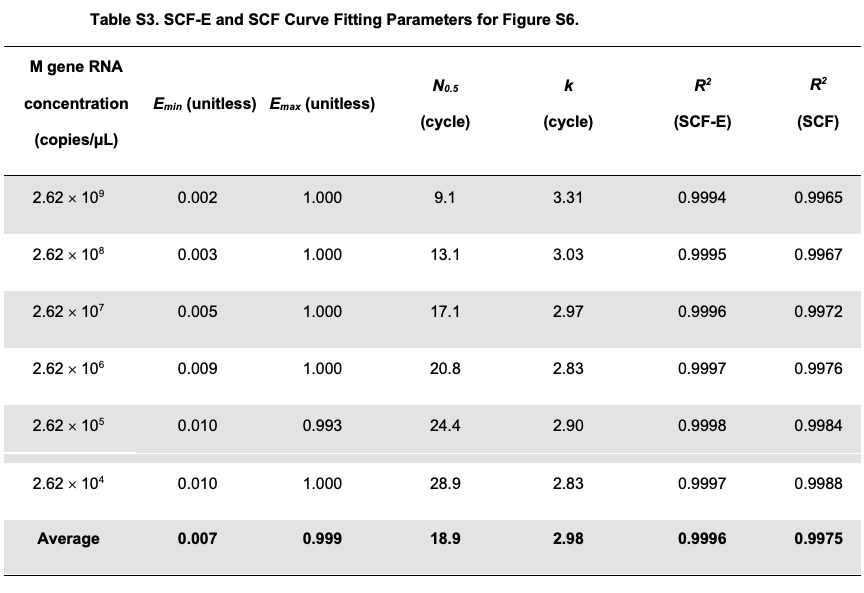

Supplement: S3 Table — (TIF) [file ppat.1012257.s045.tif]

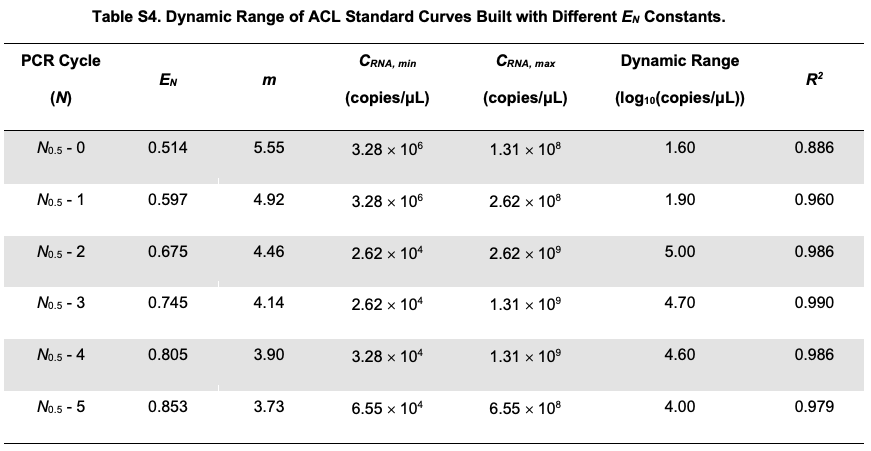

Supplement: S4 Table — We compared cycle numbers (N) at which PCR efficiency (EN) of the reference curve is used to calculate the constant m (and EN). This constant determines the RNA template concentration assigned to virtual curves in the ACL. EN is calculated using Eq. S3 and m is calculated using Eq. S13. We used the cycle number at which PCR efficiency is equal to 0.5 (N0.5) and the five cycles preceding it (N = N0.5−1 to N = N0.5−5), to construct six ACLs for comparison. From each ACL, a standard curve was generated at PCR cycle number 20. This standard curve was then used to convert fluorescence (FN) to template concentration (CRNA) for a known dilution series of M gene RNA (S2 Table, three replicates of 21 concentrations between 2.62 × 10^4^ to 2.62 × 10^9^ copies/μL). The minimum (CRNA, min) and maximum (CRNA, max) concentrations in the dilution series, whose measured RNA concentration fell within a 2-fold change of the expected concentrations, marked the dynamic range of each standard curve. The R2 value quantifies the linear regression (S8 Fig) between the measured (CRNA, ACL) and expected (CRNA, expected) RNA concentrations. The ACL built with EN at N = N0.5−3 produced a cycle 20 standard curve with the highest dynamic range and the greatest degree of accuracy (R2). Therefore, an offset of -3 cycles was used to construct all ACLs in this work. (TIF) [file ppat.1012257.s046.tif]

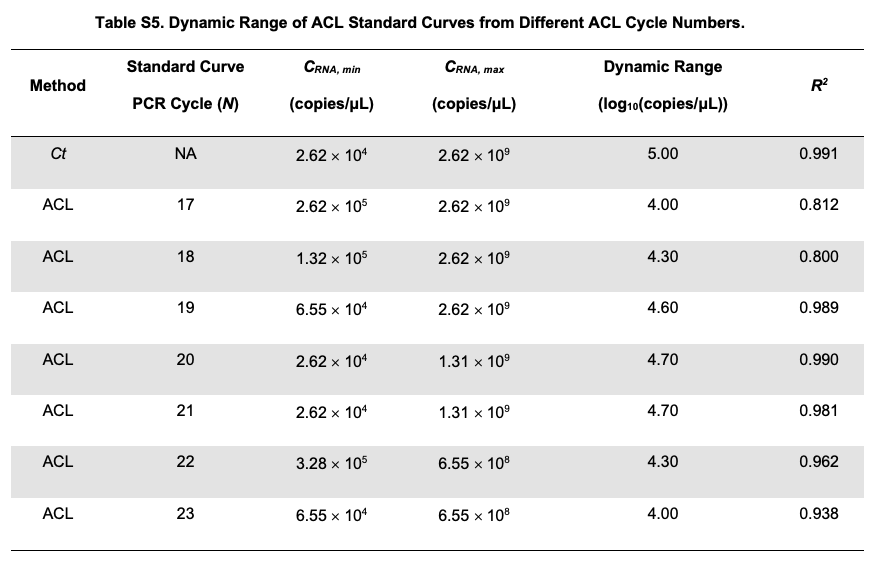

Supplement: S5 Table — We compared the dynamic range of standard curves created using the amplification curve library (ACL) method to that of the cycle threshold (Ct) method. Standard curves were used to convert fluorescence to template concentration for a known dilution series of IAV M gene RNA (S2 Table, three replicates of 21 concentrations between 2.62 × 10^4^ to 2.62 × 10^9^ copies/μL). In the ACL method, multiple standard curves were constructed from individual cycle numbers (N = 17 to 23). On the other hand, in the Ct method, a single standard curve is built from multiple Ct values, with one Ct value corresponding to each concentration of M gene RNA. The dynamic range of each standard curve was determined by identifying the minimum (CRNA, min) and maximum (CRNA, max) concentrations within the dilution series that yielded measured RNA concentration falling within a 2-fold change of the expected concentrations. The R2 value quantifies the linear response (S9 Fig) between the measured (CRNA, ACL) and expected (CRNA, expected) RNA concentrations. (TIF) [file ppat.1012257.s047.tif]

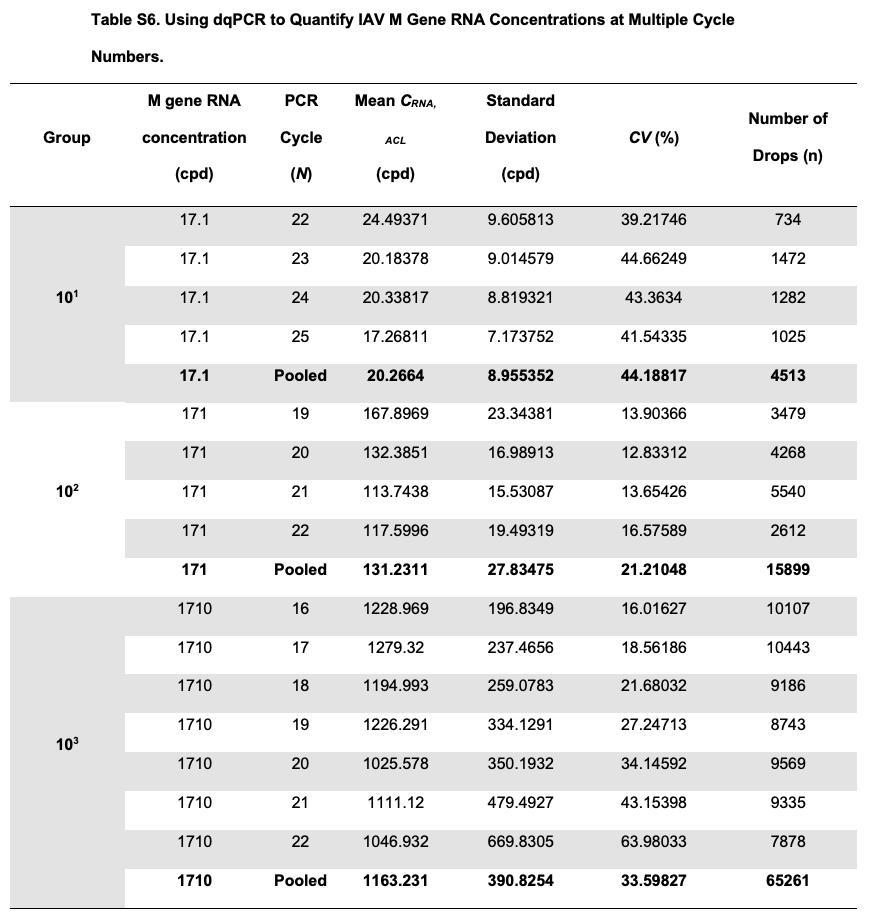

Supplement: S6 Table — Three known IAV M gene RNA concentrations, 1.71 × 101 cpd (101), 1.71 × 10^2^ cpd (102), and 1.71 × 10^3^ cpd (103), were amplified in 50 μm drops. Drop fluorescence (ΔRN) was detected at multiple PCR cycle numbers and converted to M gene cpd (CRNA, ACL) using dqPCR. CRNA, ACL measurements from different cycle numbers were pooled together and presented as a single distribution in Fig 3B. To assess the variability between individual cycle numbers and the pooled distribution, we analyzed the standard deviation of the mean CRNA, ACL and the coefficient of variation (CV %) across the number of sampled drops (n) at each N. (TIF) [file ppat.1012257.s048.tif]

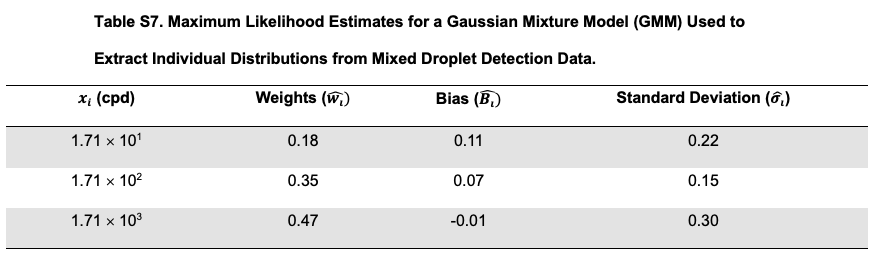

Supplement: S7 Table — To use dqPCR for measuring IAV burst size in a heterogeneous population of single-cell infections, we validated the method’s ability to isolate individual distributions from a sample containing multiple M gene RNA concentrations. We applied a Gaussian mixture model (GMM) on dqPCR data obtained from three known IAV M gene RNA concentrations: 1.17 × 101 cpd, 1.17 × 10^2^ cpd, and 1.17 × 10^3^ cpd, mixed together in a single sample (data shown in Fig 3E). Model assumptions are described in S7 Results. In the model, xi represents the number of M gene RNA copies (cpd). Each Gaussian has a different weight (wi) corresponding to the probability that a random drop in the mixture has xi copies, a predicted mean (Bi, representing measurement bias) and variance (σi2, representing measurement noise). (TIF) [file ppat.1012257.s049.tif]

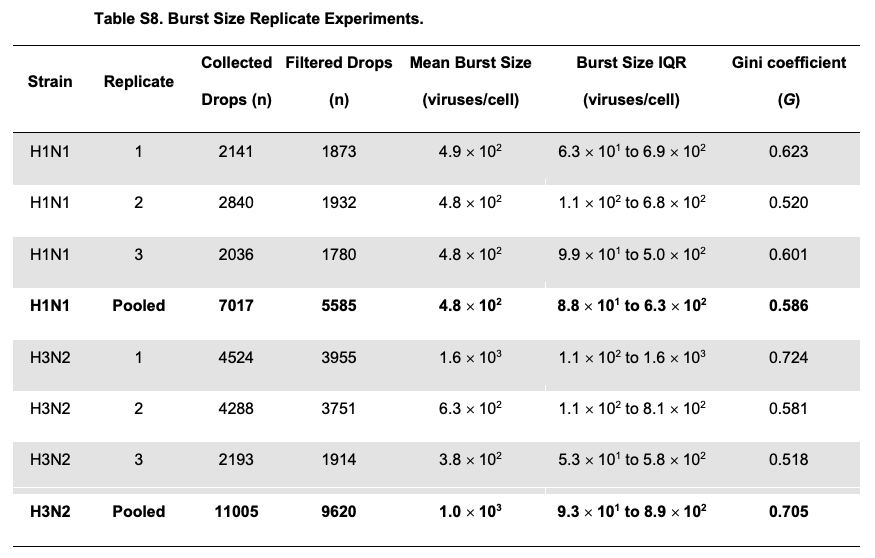

Supplement: S8 Table — IAV burst sizes were measured from thousands of individual drops sampled at cycle numbers N = 16, 19, 22, 25, and 28 in three biological replicates. The measurements were used to determine the number of IAV M gene RNA and cellular β-actin mRNA (cpd). Drops containing high β-actin cpd (S19 Fig) were assumed to contain non-packaged, intracellular, M gene RNA and were subsequently filtered from the final burst size distributions (Filtered Drops, S8 Results). We present the average measured burst size for each replicate, along with the interquartile range (IQR) that represents the lower 25th and upper 75th percentile of the distributions. The heterogeneity of each distribution was examined using the Gini coefficient (G). If G = 0, the distribution is completely homogeneous, meaning that each cell produces the same number of viruses. Conversely, if G = 1, the distribution is completely heterogeneous, where one cell produces all the viruses. Thus, a higher G corresponds to a more heterogeneous distribution. (TIF) [file ppat.1012257.s050.tif]

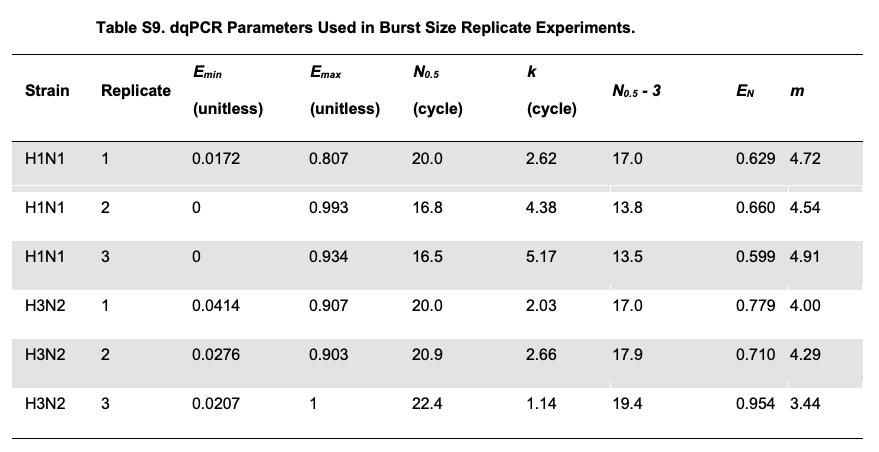

Supplement: S9 Table — The parameters used to create reference standard curves using SCF-E (S20 Fig, orange dotted curves) for each burst size experiment. (TIF) [file ppat.1012257.s051.tif]

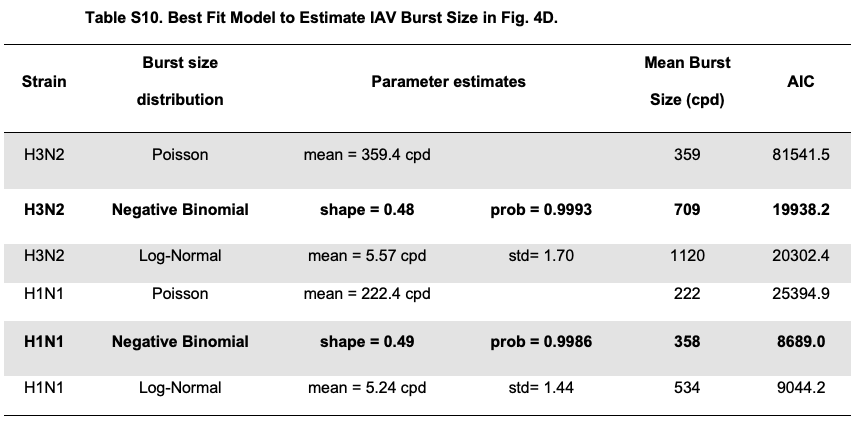

Supplement: S10 Table — We used a simulation-based approach to estimate the IAV burst distributions shown in Fig 4D. We considered three possible distributions: lognormal, Poisson, and negative-binomial, all with unknown parameters. First, we simulated the viral burst size based on the assumed distribution. For each simulated value of burst size x, we introduced measurement noise by assuming a log-normal distribution. The mean of the log-normal distribution was determined by the bias function B(x), and the standard deviation was determined by σ(x). Next, we computed a density function using a kernel density estimation with a Gaussian kernel to represent the resulting distribution. We then used this density function to calculate the log-likelihood of the observed data. We estimated the parameters for each distribution and for each dataset (H3N2 or H1N1) by maximizing the likelihood of observations reported in Fig 4D. (TIF) [file ppat.1012257.s052.tif]
